# Y-shaped trivalent aptamer for targeted visualization and tracking of reprogrammed astrocytes

**DOI:** 10.1016/j.mtbio.2025.102482

**Published:** 2025-10-28

**Authors:** Bohyun Oh, Eun-Song Lee, Eun-Hye Lee, Kyung-Min Kim, Yeonju Lee, Jin-Sam Lee, Hong-Gyun Lee, Hyobin Jeong, Chang-Hwan Park, Young-Pil Kim

**Affiliations:** a Department of Life Science, Hanyang University, Seoul, 04763, Republic of Korea; b Neuroregeneration and Stem Cell Programs, Institute for Cell Engineering, Johns Hopkins University School of Medicine, Baltimore, MD 21205, USA; c Department of Neurology, Johns Hopkins University School of Medicine, Baltimore, MD 21205, USA; d Hanyang Biomedical Research Institute, Hanyang University, Seoul, 04764, Republic of Korea; e School of Biological Sciences, Seoul National University, Seoul, 08826, Republic of Korea; f Department of Systems Biology, College of Life Science and Biotechnology, Yonsei University, Seoul, 03722, Republic of Korea; g Bureau of Research & Development Innovation, Korea Health Industry Development Institute, Cheongju, 28159, Republic of Korea; h Research Institute for Convergence of Basic Science & Research Institute for Natural Sciences, Hanyang University, Seoul, 04763, Republic of Korea; i Hanyang Institute of Bioscience and Biotechnology, Hanyang University, Seoul, 04763, Republic of Korea

**Keywords:** Astrocyte, Trivalent, SELEX, Aptamer, Transdifferentiation, Glioblastoma, Targeted imaging

## Abstract

Despite extensive research into the diverse transformations and functions of astrocytes, conventional fluorescence immunohistochemistry for their distinct identification remains challenging and time-consuming, primarily due to the lack of cell surface binders specific to these glial cells. To address this limitation, we developed a specific and straightforward imaging strategy for primary astrocytes using cell surface-targeting aptamers. We identified a novel anti-astrocyte DNA aptamer (designated Ast17-30) through a 17-round cell-SELEX process incorporating magnetic-activated cell sorting, designed to bypass the technical hurdles of SELEX when applied to short-lived cells. To enhance binding affinity, we further engineered this aptamer into a Y-shaped trimer (Tri-ΔAst17-30), enabling clear discrimination between astrocytes and neurons. Consequently, the engineered trivalent aptamer facilitated rapid astrocyte-specific endocytosis within minutes due to its increased binding avidity. This capability enabled longitudinal monitoring of astrocyte conversion into induced neural precursor cells, observing moderate pro-inflammatory gene expression in their transcriptomic profiles. Furthermore, Tri-ΔAst17-30 bound strongly to astrocytic glioblastoma cells (U87MG), with minimal binding to glial glioblastoma cells (C6), confirming specificity for astrocytic tumor cells. Given the current absence of reliable live-astrocyte-specific imaging techniques, we propose that this trivalent anti-astrocyte DNA aptamer has potential for investigating developmental pathways and targeted therapy of these crucial glial cells.

## Graphical abstract

**Figure:**
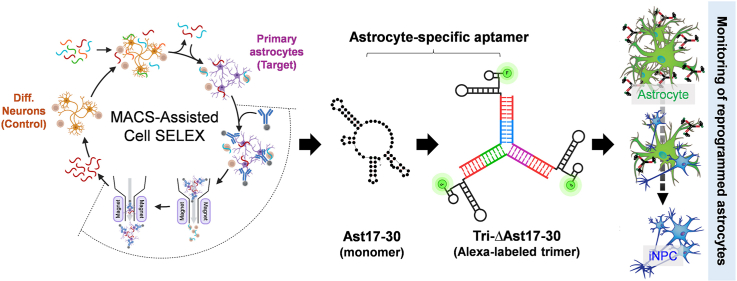

## 1 Introduction

Star-like astrocytes, the most prevalent glial cells in the central nervous system, exhibit significant heterogeneity and play critical roles in brain function by modulating nutrient metabolism, neuronal activity, cerebral blood flow, and neuroinflammatory processes [[1], [2], [3]]. The reprogramming of astrocytes in response to both inter- and intracellular cues is increasingly recognized as a promising therapeutic strategy for neurodegenerative diseases [[4], [5], [6]]. To fully elucidate the diverse or therapeutic functions of astrocytes, the development of a rapid and effective tool for monitoring and tracking their differentiation dynamics is essential. However, despite considerable efforts to image astrocytes, the sequential visualization of their dynamic changes during transdifferentiation remains a significant challenge, largely attributable to the absence of cell surface biomarkers uniquely specific to these cells. Traditional immunopanning or immunohistochemistry (IHC) methods for astrocyte identification have relied on antibodies targeting cognate surface epitopes, intermediate filament glial fibrillary acidic protein (GFAP), astrocytic glutamate transporter (GLT), Ca^2+^-binding protein (S100β), or aldehyde dehydrogenase 1 family member L1 (ALDH1L1) [7,8]. Moreover, astrocytes expressing reporter genes under the control of specific promoters have been employed in fluorescence-activated cell sorting (FACS) or *in vivo* imaging [[9], [10], [11], [12]]. Nevertheless, these antibody- and gene reporter-based approaches target cytosolic and nuclear proteins, rendering them unsuitable for monitoring naïve astrocytes. While antibodies targeting astrocyte cell surface antigen 1 (ACSA-1) and 2 (ACSA-2), which recognize an extracellular domain of glutamate aspartate transporter (GLAST), have been used to characterize viable astrocytes or radial glial cells within the astrocyte lineage [13,14], their reliance on single epitope specificity may not ensure isolation of a homogeneous astrocytic population. For instance, the regional and subcellular expression levels of GLAST have been shown to vary across astroglia cells as well as neurons [15]. Importantly, the antibody-based approach is generally neither straightforward nor ideal for intracellular delivery, primarily due to the inherent limitations associated with their large molecular size.

A promising strategy to overcome these limitations involves the use of aptamers, which can be efficiently selected from large combinatorial libraries via cell-based systematic evolution of ligands by exponential enrichment (SELEX) process [16,17]. Even in the absence of prior knowledge regarding cell surface proteins, a diverse panel of aptamers can be identified through iterative negative and positive SELEX rounds using target and non-target cells, respectively. In comparison to antibodies, nucleotide-based aptamers possess several advantages, including compact size, ease of production and labeling, modular engineering capabilities, and robust stability while retaining target specificity [[18], [19], [20]]. Despite these benefits, the development of astrocyte-targeting aptamers remains unexplored, likely due to astrocytic heterogeneity and the technical difficulties of stringent counter-selection. We hypothesized that differential screening between primary astrocytes and their reprogrammed derivatives could yield aptamers capable of distinguishing astrocytes in stem-like lineage processes, even in the absence of comprehensive data on astrocytic surface receptor composition. While numerous studies have monitored transcription factor- or small molecule-induced astrocyte reprogramming using genetically encoded reporters or antibody-based IHC [[21], [22], [23]], these approaches would benefit from a faster, simpler methodology compatible with live astrocytes.

Herein, we report the discovery and engineering of a single-stranded (ss) DNA aptamer for specific identification of primary astrocytes. This anti-astrocyte aptamer enables both the targeted imaging of astrocytes and the visualization of their transdifferentiation into neuronal lineages. To achieve this, highly diverse ssDNA libraries, including a 40-nucleotide randomized region, underwent iterative SELEX rounds using rat primary cortical astrocytes (positive selection) and neurons differentiated from rat cortical neural progenitor cells (NPCs) (negative selection). By performing concurrent negative-positive selection in each round, we enriched an aptamer library with enhanced specificity for astrocytes over differentiated neurons. We integrated the cell SELEX procedure into magnetic-activated cell sorting (MACS) conjugated with an anti-GLAST antibody to eliminate GLAST-negative or non-viable cells. To boost binding affinity, we engineered selected aptamers into a Y-shaped trivalent configuration using a self-assembled DNA-origami scaffold. This Y-shaped configuration was strategically chosen to maximize multivalent binding while minimizing steric hindrance. It provides optimal spatial alignment for engaging clustered cell-surface targets, striking a practical balance between increased avidity and manageable structural complexity. In contrast, constructs with four or more arms can experience reduced efficiency due to steric crowding and geometric mismatches with cellular epitopes, as supported by both computational and experimental studies [24]. Subsequently, the engineered aptamer underwent rigorous evaluation to ascertain its ability to monitor transdifferentiation dynamics and selectively target astrocytic glioblastoma using complementary fluorescence (FL) imaging and FACS analysis platforms.

## 2 Material and methods

### 2.1 Primary rat astrocyte cultures

The animals were housed and treated according to the Institutional Animal Care and Use Committee (IACUC) guidelines of Hanyang University, Korea. After removal of the meninges, cerebral cortical tissue from Sprague Dawley (SD) rats (DaeHan BioLink, Seoul, Korea) at postnatal day 5 (P5) was dissected and dissociated mechanically. Then, cells were plated on T-75 flasks and suspended in a Dulbecco's Modified Eagle Medium/Nutrient Mixture F12 (DMEM/F12; Thermo Fisher Scientific, Waltham, MA, USA) supplemented with 10 % fetal bovine serum (FBS; Thermo Fisher Scientific), 8 % horse serum (HS; Thermo Fisher Scientific), 50 mM glucose (Sigma-Aldrich, St. Louis, MO, USA), penicillin/streptomycin (Thermo Fisher Scientific), B27 (Thermo Fisher Scientific), Glutamax (Thermo Fisher Scientific), and 20 ng mL^−1^ basic fibroblast growth factor (bFGF; R&D Systems, Minneapolis, MN, USA). When cell confluence reached 90 %, minor microglia were removed by gentle shaking, pure astrocytes were harvested and replated in a dish coated with poly-L-ornithine (PLO; 15 μg mL^−1^, Sigma-Aldrich) and fibronectin (FN; 1 μg mL^−1^, Sigma-Aldrich). For astrocyte differentiation, FBS, HS and bFGF were withdrawn from the medium and 10 ng mL^−1^ ciliary neurotrophic factor (PeproTech, Rocky Hill, NJ, USA) was added.

### 2.2 Primary rat neural progenitor cell (NPC) cultures and neuronal differentiation

After removing the embryos from female SD rats at embryonic day 14 (E14), the cerebral cortical tissue was dissected and single cells were isolated from the tissue. NPCs were seeded on 10 cm culture dishes coated with PLO/FN and the growth medium was composed of a serum-free medium (N2) to which 20 ng mL^−1^ bFGF was added. For neuronal differentiation, the medium was changed to neurobasal medium (Thermo Fisher Scientific) supplemented with penicillin/streptomycin, B-27, and Glutamax.

### 2.3 SELEX procedure

The combinatorial ssDNA library and modified ssDNA were synthesized by Integrated DNA Technologies Inc. (IDT, Coralville, IA, USA). Aptamers and primers were synthesized by Macrogen Inc. (Seoul, Korea). A SELEX process was performed to select ssDNA aptamers binding specifically to primary rat astrocytes, which encompassed successive steps of negative and positive SELEX using target cells (primary astrocytes) or counter-target cells (primary neurons) in every round. For binding primary cells with ssDNA library, cultured primary cells (astrocytes or neurons) on 100-mm sterile dishes were dissociated using 1 × Accutase (Thermo Fisher Scientific) for 5 min at 37 °C in a humidified 5 % CO_2_ incubator immediately after removing culture medium. The cells were then collected in 15 mL Falcon tubes (SPL Life Sciences, Pocheon, Korea) by centrifugation at 1000×*g* for 1 min at room temperature (RT). After washing twice in washing buffer (Dulbecco's phosphate-buffered saline; DPBS containing 1 % glucose and 1 mM MgCl_2_), cells were resuspended at 500 μL (neurons) or 100 μL (astrocytes) of binding buffer (DPBS containing 4.5 % glucose, 100 mg mL^−1^ BSA, 100 mg mL^−1^ salmon sperm DNA, and 5 mM MgCl_2_) in 1.5 mL Eppendorf tubes. Unless otherwise stated, the number of viable cells was assessed using trypan blue dye exclusion in a hemocytometer. In the negative cell-SELEX, 500 μL of solution containing differentiated neurons at a density of 2.5–5 × 10^6^ cells mL^−1^ was incubated with ssDNA library (500 μL at appropriated concentrations) dissolved in the binding buffer for 30−60 min at 4 °C. The ssDNA-containing supernatant was collected by centrifugation at 300×*g* for 10 min at 4 °C. In the next positive SELEX, 900 μL of the supernatant was mixed with 100 μL of the solution containing Accutase-treated astrocytes at a density of 2.5–5 × 10^6^ cells mL^−1^ for 10−60 min at 4 °C. Next, ssDNA library-bound cells were rinsed three times in washing buffer by centrifugation at 300×*g* for 10 min at 4 °C to remove unbound ssDNA, resuspended in 500 mL of distilled water, and subjected to boiling at 95 °C for 15 min. The ssDNA library was then collected from the supernatant by centrifugation (13,100×*g* for 10 min at RT) and eluted from cell debris. To amplify eluted ssDNA library, polymerase chain reaction (PCR) was performed using a ProFlex Thermocycler (Thermo Fisher Scientific) in a two-step linear manner. The initial PCR was conducted in 50 μL reactions containing 23 μL of ssDNA library (supernatant in DW), 25 μL of 2 × Hot Start GoTaq master mix (Promega, Madison, WI, USA), 1 μL of 25 μM forward primer, and 1 μL of 25 μM reverse primer, followed by five cycles of 95 s at 4 °C (denaturation), 30 s at 4 °C (annealing), and 20 s at 72 °C (extension). The second PCR was conducted in 50 μL reactions containing 2 μL of the 1st PCR product, 25 μL of 2 × Hot Start GoTaq master mix, 21 μL of distilled water, 1 μL of 25 μM forward primer, and 1 μL of 25 μM reverse primer at 3−25 cycles. The final PCR product (double-stranded DNA; dsDNA) was monitored using 3 % agarose gel electrophoresis (100 V, 30 min) and purified using an agarose gel cleanup kit (Qiagen, Hilden, Germany). To generate the ssDNA library, asymmetric PCR was carried out in 50 μL reactions containing dsDNA template, Hot Start GoTaq master mix, and forward/reverse (10:1 M ratio) primers, followed by 15–30 cycles of 95 s at 4 °C (denaturation), 30 s at 4 °C (annealing), and 20 s at 72 °C (extension). The ssDNA library was purified using an agarose gel cleanup kit and a gel extraction bead (Qiagen). The agarose gel was imaged and analyzed using a home-built transilluminator equipped with a blue light-emitting diode (LED) lamp. The concentration of ssDNA library was determined using a NanoDrop spectrophotometer (Colibri Microvolume; Berthold Technologies, Pforzheim, Germany). The purified product was stored at 4 °C prior to use in the next round. Negative and positive cell SELEXs were repeated alternately from 1 to 13 rounds, whereas the magnetic assisted cell sorting (MACS) process was introduced into the positive SELEX from 14 to 20 rounds to increase astrocyte-specific binding and minimize nonspecific interactions. For MACS, anti-ACSA-2 MicroBead Kit (Miltenyi Biotec, Bergisch Gladbach, Germany) was used to select GLAST-positive astrocytes based on a magnetic cell sorting technique provided by the manufacturer. Briefly, 20 μL of biotinylated anti-GLAST antibody was incubated with 80 μL of aptamer library-treated astrocytes for 10 min at 4 °C, followed by incubation of anti-biotin antibody-conjugated magnetic beads for 10 min. The ssDNA-bound astrocytes were rinsed in PBS buffer containing 0.5 % BSA using an LS column (Miltenyi Biotec) under the magnetic field, and the beads were eluted from the column upon magnet removal. The ssDNA library was harvested from the beads using thermal treatment and centrifugation, followed by the same protocol mentioned above. In addition to MACS, a set of reaction conditions including cell number, binding time, and aptamer library concentration was stringently controlled in the negative or positive cell-SELEX (Table S1). Quantitative real-time PCR (qPCR) was performed to monitor the aptamer library enrichment using a CFX connect real-time system (Bio-Rad, CA, USA) with TB green (Takara, Kyoto, Japan).

### 2.4 Aptamer sequencing

Following the generation of a saturated library pool from qPCR, ssDNA eluates from rounds 12, 13 and 17 were amplified into dsDNA pools using Taq polymerase (Promega, Madison, WI, USA). The purified dsDNA was subcloned into a T-vector (Promega) for full-length sequence retrieval; the plasmid was transformed into *E. coli* DH5α, incubated at 37 °C under antibiotic selection, and 40−60 positive colonies from each round were randomly selected for sequencing. Multiple alignments of selected aptamers were performed using ClustalX2 to analyze group similarity and secondary structures of aptamer candidates were predicted using the Mfold web server.

### 2.5 Preparation of trivalent aptamers

A Y-shaped trimeric aptamer was prepared by self-assembly of three ssDNA fragments (Ya, Yb, and Yc), as reported previously [25]. Briefly, equimolar mixtures (20 μM each) of the three strands in 50 mM phosphate buffer (containing 100 mM NaCl, pH 8.0) were denatured at 95 °C for 2 min and annealed to 4 °C at a rate of 1 °C min^−1^ to generate a three-armed Y-scaffold with eight-base sticky ends. This scaffold was then mixed with 60 μM of Alexa488-labeled tAst17-30 ssDNA aptamer (3′ end labeled) and incubated at 20 °C for 2 h to form Y-shaped trimeric aptamers. The preparation and assembly of both the Y-shaped DNA scaffold and the trivalent aptamers were characterized by 0.5 % agarose gel electrophoresis in 1 × TBE buffer for 1 h, followed by imaging and analysis using a custom gel documentation system.

### 2.6 Generation of iNPCs from primary rat astrocytes

Primary rat astrocytes were transduced overnight with retrovirus particles encoding *Brn2, Ascl1, Myt1L* and *Bcl-XL* [26]. After 48 h, the medium was replaced with neural induction medium, N2 supplement containing 20 ng mL^−1^ bFGF, 100 U mL^−1^ recombinant human leukemia inhibitory factor (Millipore, Temecula, CA, USA) and 2 μg mL^−1^ doxycycline (Sigma-Aldrich). At day 7, cells were replated onto PLO/FN-coated dishes and maintained in induction medium for 3–4 weeks. Upon observation of dense cell clusters, these were manually isolated and dissociated into single-cell iNPC suspensions using Accutase (Millipore).

### 2.7 qPCR and reverse transcription quantitative PCR (RT-qPCR)

For ssDNA aptamer library quantification, aptamer-bound primary cells were harvested after 5 min 1 × Accutase treatment at 37 °C, washed, and suspended in 500 μL distilled water as qPCR templates. For mRNA analysis, total RNA extracted with TRI reagent (Molecular Research Center Inc., Cincinnati, OH, USA) was reverse-transcribed with Superscript kit (Thermo Fisher Scientific) and amplified using gene-specific primers in triplicate qPCR reactions on a CFX96 Real-Time System with iQ SYBR green supermix (Bio-Rad, Hercules, CA, USA). For RT-qPCR, the target gene expression was normalized to glyceraldehyde 3-phosphate dehydrogenase (*Gapdh*) expression. The following primers were used: *Gapdh*-F: 5′−GGC ATT GCT CTC ATT GAC AA−3′, *Gapdh*-R: 5′−AGG GCC TCT CTC TTG CTC TC−3′, *Nestin*-F: 5′−CTG AGG CCT CTC TTC TTC CA −3′, *Nestin*-R: 5′− ACT CCT GTA CCG GGT CTC CT−3′, *Sox2*-F: 5′−GCG ACC GGC GGC AAC CAG AAG AAC−3′, *Sox2*-R: 5′− GCC GGC GCC CAC CCC AAC C−3′, *Gfap*-F: 5′−GCA GAC CTC ACA GAC GTT GCT−3′, and *Gfap*-R: 5′−AGG CTG GTT TCT CGG ATC TGG−3′.

### 2.8 Confocal imaging

For imaging analysis, 10 mm rounded coverslips were placed into 24-well plates prior to cell culture. Cultured primary cells were then washed twice with PBS and blocked with 3 mg mL^−1^ dextran sulfate for 10 min at 37 °C. Alexa488-labeled aptamers, diluted in binding buffer, were incubated with cells for 1 h in the dark at 37 °C. Unbound aptamers were removed through two washes with washing buffer. For IHC, cells were fixed with 4 % paraformaldehyde. They were subsequently blocked and permeabilized for 1 h at RT using a solution of 10 % normal goat serum, 0.1 % BSA, and 0.3 % Triton-X 100. For immunostaining, primary antibodies were incubated overnight at 4 °C, followed by a 1 h incubation with FL-labeled secondary antibodies for 1 h at RT. The following primary and secondary antibodies were used: glial fibrillary acidic protein (GFAP; DAKO, Glostrup, Denmark), TUJ1 (Covance, Richmond, CA, USA), Nestin (BD, #556309, USA), Cy3-labeled anti-mouse antibody (Jackson Immuno Research, #115-165-146, USA), and Cy5-labeled anti-goat antibody (Thermo Fisher Scientific, #2063326). Nuclei were stained with 4′,6-diamidino-2-phenylindole (DAPI) in Vectashield mounting medium (Vector Laboratories, Burlingame, CA, USA). Confocal microscope images were acquired using a Nikon C2si (Tokyo, Japan).

### 2.9 FACS analysis

Primary cells were prepared by incubating them with 1 × Accutase for 5 min at 37 °C. Following two washes with PBS, cells were blocked with 3 mg mL−1 dextran sulfate for 10 min at 4 °C. Subsequently, they were incubated with ALEXA488-labeled aptamers for 30−120 min at 4 °C. Unless otherwise stated, the final concentration of Alexa488-conjugated aptamers in confocal and FACS analyses was 500 nM based on monomeric units; that is, 500 nM for Ast17-30 and ΔAst17-30, and 167 nM for Tri-ΔAst17-30. After two additional washes with PBS, cells were filtered through a cell strainer prior to FACS acquisition. Flow cytometry was performed on a BD FACS Canto II or ARIA III (BD Biosciences, USA), and data were analyzed using Flow Jo software (BD Biosciences, USA). Gates were established based on unstained controls. Debris and cell clumps were effectively discriminated by applying forward scattering (FSC) and side scattering (SSC) gating. To estimate the binding affinity of the aptamers, we analyzed FACS data based on the percentage of aptamer-positive cells in primary astrocyte population. For normalization, the background signal from a negative control group (differentiated neurons) was subtracted at each aptamer concentration. Aptamer concentrations were displayed based on their monomeric units (i.e., the final concentration of Tri-ΔAst17-30 is three times that of monomeric ΔAst17-30). The apparent dissociation constant (*K*_d_^app^) was derived by non-linear regression of the normalized binding (%) curve according to the following equation (Eq. (1)):

(Eq. 1) $$\text{Normalized}\;\text{Binding}\;\left(\%\right)=100\times \left(\frac{\text{Binding}{\left[C\right]}_{\text{Ast}}-\text{Binding}{\left[C\right]}_{\text{Neu}}}{{\text{Binding}}_{\mathrm{MAX}\;}-\text{Binding}{\left[C\right]}_{\text{Neu}}}\right)$$

where Binding[C]_Ast_ is the percentage of aptamer-positive primary astrocytes at a given aptamer concentration, Binding[C]_Neu_ is the percentage of aptamer-positive differentiated neurons at a given aptamer concentration (representing nonspecific background binding), and Binding_MAX_ denotes the maximal aptamer-binding percentage.

### 2.10 Fluorescent imaging glioblastoma cells using aptamers

Human astrocytic glioblastoma U-87MG and rat glial glioblastoma C6 cell lines were acquired form the Korean Cell Line Bank (Seoul, Korea). Both cells were cultured in T-75 cell culture flask in DMEM supplemented with 10 % FBS, 100 U/mL penicillin, and 100 μg/mL streptomycin. Cells were maintained in a humidified incubator at 37 °C with 5 % CO_2_ until they reached 80−90 % confluence. For confocal imaging, cells were incubated with Alexa488-conjugated ΔAst17-30 and Tri-ΔAst17-30 aptamers in serum-free DMEM at final concentrations of 200 nM and 66 nM, respectively, for 1 h at 37 °C in the dark to allow for binding. Unbound aptamers were removed by washing the cells twice with 400 μL of 1 × DPBS. Cells were then fixed with 200 μL of 4 % paraformaldehyde for 10 min at RT, followed by incubation in 400 μL of 0.1 M glycine for 10 min at RT. After two final washes with 1 × DPBS, cells were mounted with Vectashield HardSet antifade medium containing DAPI for nuclear staining. Samples were sealed with a cover glass and stored overnight at 4 °C. Imaging was performed using a Zeiss LSM 900 confocal laser scanning microscope. Images were acquired via sequential scanning, with excitation wavelengths of 405 nm for DAPI and 488 nm for Alexa488. Emission ranges were 400–515 nm and 400–650 nm, respectively. For the measurement of intracellular reactive oxygen species (ROS), U87MG cells were subcultured into 96-well plates and cultured in DMEM supplemented with 10 % FBS and 1 % penicillin-streptomycin. The cells were then three times with phenol red-free DMEM (containing 1 % P/S without FBS) before being treated with Tri-ΔAst17-30 at various concentrations (50 nM−1 μM). Following a 24-h incubation at 37 °C, the cells were washed again three times. Subsequently, the cells were incubated with 10 μM CellROX reagent (Thermo Fisher Scientific) for 30 min at 37 °C to stain for intracellular ROS. After a final wash with 1 × DPBS, FL intensity was measured using a plate reader at 490/520 nm.

### 2.11 Singe-cell RNA sequencing and data processing

Cerebral cortical tissues from SD rats underwent mechanical dissection and dissociation for subsequent scRNA-seq analysis. The resulting cells were cultured on two 100 mm petri dishes in DMEM/F12, enriched with 10 % FBS, 8 % HS, 50 mM glucose, penicillin/streptomycin, B27, Glutamax, and 20 ng/mL bFGF. Without allowing further growth, these primary cells from one culture dish were then exposed to 200 nM trivalent aptamer (Tri-ΔAst17-30) in their culture media and incubated for 1 h at 4 °C. To generate a single-cell preparation, the cells were treated with 1 × Accutase for 5 min at 37 °C. After a single centrifugation wash with fresh culture media, the primary cells were resuspended in 200 mL of culture media, immediately transferred to ice, and subsequently used for sequencing analysis. Raw scRNA-seq data, encompassing feature, barcode, and matrix files for both non-aptamer-treated (noApt) and aptamer-treated (withApt) groups, underwent processing using Seurat (v4.3.0) within R (v4.2.2). The data were initially loaded via the Read10 × () function to establish a Seurat object, followed by a quality control regimen that retained only cells with more than 750 detected genes (nFeature_RNA >750) and more than 1000 total RNA counts (nCount_RNA >1000), while simultaneously excluding potential doublets by removing cells with RNA counts exceeding two standard deviations above the mean (nCount_RNA < μ + 2σ), and filtering out cells with mitochondrial transcript fractions greater than 20 % (percent.mito >20). Subsequently, data integration was performed using FindIntegrationAnchors() and IntegrateData() with the initial 20 principal components, which then led into data scaling (ScaleData()) and principal component analysis (RunPCA()). Principal components 1–10 were precisely selected based on insights from elbow plot inspection for subsequent analytical steps. For clustering and visualization, a nearest neighbor graph was constructed (FindNeighbors()), followed by cell clustering at a resolution of 0.4 (FindClusters()), and UMAP visualization (RunUMAP()). Finally, manual cell type annotation was executed for each cluster, leveraging canonical marker genes indicative of astrocytes, oligodendrocytes, neurons, microglia, fibroblasts, and oligodendrocyte precursor cells (OPCs). These annotations were validated through FeaturePlot() visualization and differential gene expression.

### 2.12 Differential expression analysis and visualization

We performed differential expression analysis for each of the identified clusters (0−9). To visualize expression differences between the "noApt" and "withApt" groups, we first computed the average gene expression per group using AverageExpression() and then log-transformed the values (log1p). Scatter plots were generated using ggplot2, and key genes of interest were labeled with LabelPoints() from the cowplot package, employing customized repelling and text size parameters for clarity. This process was repeated across all clusters to assess condition-specific expression patterns. For a more in-depth examination, differential gene expression and volcano plot visualization were specifically conducted for clusters 0, 2, 8, and 9. Cells from these particular clusters were subset from the integrated dataset. Before analysis, the default assay was set to "RNA," and layers were merged using JoinLayers() to facilitate differential expression calculations. Cells were then grouped based on the "apt" metadata label (withApt vs. noApt). We identified differentially expressed genes using FindMarkers() with a minimum expression threshold (min.pct = 0.05) and a log-fold change threshold (logfc.threshold = 0.25). The resulting gene lists were subsequently visualized using EnhancedVolcano, effectively highlighting genes that were significant based on adjusted p-values and log_2_ fold changes.

### 2.13 Pathway module scoring

To explore condition-dependent signaling activity, we calculated two types of expression-based module scores: ligand-receptor module scores and TNF-NF-κB pathway scores. For ligand-receptor analysis, we built custom gene modules based on established interactions, including *Ccl3–Ccl5, Ccl3–Ccr1, Ccl4–Ccr5, and Cxcl2–Cxcr2*. We then computed the average normalized expression for each gene pair using MatrixcolMeans() on log-transformed RNA assay data, storing these scores as metadata within the Seurat object. To assess TNF-NF-κB signaling, we curated a gene set of key canonical pathway components: *Tnf, Tnfrsf1a, Ripk1, Tradd, Traf2, Traf5, Tab1, Tab2, Tab3, Map3k7, Chuk, Ikbkb, Ikbkg, Nfkbia, Nfkb1*, and *Rela*. Any listed genes that were not actually detected were excluded. Using Seurat's AddModuleScore() function, we calculated a pathway activity score (TNF_NFKB_linear1) for each cell based on the remaining valid genes. Subsequently, we extracted cells from astrocyte-enriched clusters (clusters 0, 2, 8, and 9) and grouped them by treatment condition ("noApt" vs. "withApt"). We then compared module scores for ligand-receptor interactions and the TNF-NF-κB pathway using Wilcoxon rank-sum tests. For visualization, we used violin plots (ggpubrggviolin()) with jittered points and annotated *p*-values to clearly show differences in signaling activity between the experimental groups.

### 2.14 Statistical analysis

Statistical analysis was performed using the SPSS software program (IBM, ver. 26.0). Statistical significance was determined by Student's *t*-test or one-way ANOVA, as specifically indicated in the figure legends. Where applicable, all datasets underwent normality testing via the Shapiro-Wilk test. For normal distributions, Student's *t*-test or one-way ANOVA with Scheffe's post-hoc test was performed only if the data were truly normal. *N*-values represent the sample size and *p*-values are denoted as ∗*p ≤* 0.05, ∗∗*p* ≤ 0.01, ∗∗∗*p* ≤ 0.001, and ∗∗∗∗*p* ≤ 0.0001. For comparisons involving more than two groups, a paired *t*-test was conducted using Prism (ver. 8.4.3, GraphPad Software, MA, USA) or SigmaPlot for Windows (ver. 10.0, Systat Software GmbH, Erkrath, Germany).

## 3 Results and discussion

### 3.1 Discovery of anti-primary astrocyte ssDNA aptamers

**Fig. 1:**
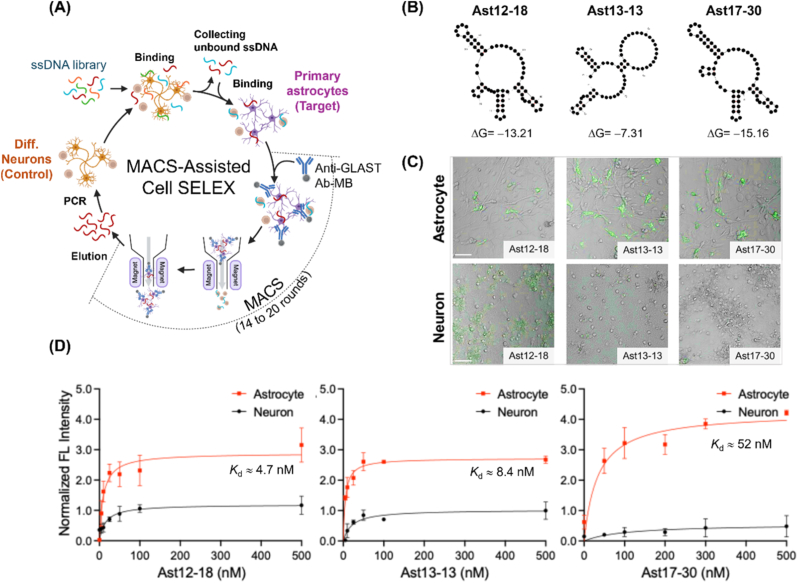
Discovery of anti-astrocyte ssDNA aptamers. **(A)** Schematic illustration of MACS-assisted cell-SELEX for aptamer selection targeting astrocytes. This iterative process involved sequential rounds of binding an ssDNA library to target rat primary astrocytes (positive-SELEX) and counter-selection against differentiated neurons (negative-SELEX). Anti-GLAST-antibody-conjugated MACS was employed from rounds 14 to 20. **(B)** Predicted secondary structures and minimum free energy (ΔG) values of the three selected aptamers: Ast12-18, Ast13-13, and Ast17-30. **(C)** Overlay images of bright-field and FL demonstrating the binding of Alexa488-labeled aptamers (green) to primary astrocytes and NPC-differentiated neurons. Scale bar, 50 μm. **(D)** Binding affinity curves of the three selected aptamers to primary astrocytes (closed red circles) and differentiated neurons (closed black circles) determined by qPCR. Normalized FL intensity is plotted against aptamer concentration, with dissociation constant (*K*_d_) for astrocyte binding. Error bars represent standard deviations (±SD) from three independent experiments. (For interpretation of the references to color in this figure legend, the reader is referred to the Web version of this article.)

As depicted in Fig. 1A, a monolayer of differentiated neurons, initially seeded at a density of 5 × 10^6^ cells, served as the counter-target for *in vitro* selection from an ssDNA library (comprising a 40-mer random region flanked by two 15-mer primer sequences), and primary astrocytes at the same cell density were used as the target in each round. Primary astrocytes were isolated and cultured from rat brain cortex, whereas neurons were differentiated from primary rat cortical NPCs. The differentiation was necessary because naïve rat neurons exhibited limited availability and a short lifespan, rendering from them unsuitable for direct cell-SELEX application. Despite inherent heterogeneity arising from their *in vivo* origin or stem cell differentiation, the two cell types exhibited distinct morphologies and strong expression of their classical markers: GFAP for astrocytes and TUJ1 for neurons (Fig. S1). To prevent the loss of cell receptor-bound aptamers, adherent cells were harvested by scraping the flask bottom without trypsin treatment. The ssDNA library in the cell-SELEX procedure underwent 13 iterative rounds of cell-binding, enrichment, and amplification without MACS, which contributed to the enhanced diversity of aptamer pools. From rounds 14 to 20, anti-GLAST antibody-conjugated MACS was integrated into the cell-SELEX procedure to enhance astrocyte-specific binding and minimize non-specific interactions (Table S1). This incorporation led to a significant enrichment of astrocyte-binding aptamer pools compared to the initial and round-13 pools when the enrichment of the ssDNA aptamer library in each round was monitored by absorbance measurements or qPCR to estimate the total amount of aptamer pools (Fig. S2). Heterogeneous astrocytes have been reported to predominantly express GLAST on their cell membranes due to their role in de novo glutamate synthesis in the brain [27,28]. To assess the effect of MACS on cell-SELEX, sequences from clonal sets at rounds 12, 13, and 17 were analyzed based on library enrichment and categorized into putative families by consensus motif alignment. Three aptamers (Ast12-18, Ast13-13, and Ast17-30) were selected based on clonal dominance and stable free energy (Fig. S3 and Table S2), representing distinct hammerhead structures with unique satellite hairpins (Fig. 1B). Ast12-18 and Ast17-30 have the same sequence except for one nucleotide, but their secondary structures differ in a small bulge region. To confirm target specificity, rat primary astrocytes and differentiated neurons were incubated with the three Alexa488-labeled aptamers, and confocal microscopy revealed stronger FL intensity in astrocytes for all three aptamers, although strong signal from Ast12-18 was also observed in differentiated neurons (Fig. 1C). While all three aptamers exhibited higher binding affinities for astrocytes (4.7, 8.4, and 52 nM in Ast12-18, Ast13-13, or Ast17-30, respectively) compared to differentiated neurons as determined by qPCR, Ast17-30 showed the highest target-to-non-target signal ratio at equivalent aptamer concentrations (Fig. 1D). Notably, the aptamers selected without MACS (Ast12-18 and Ast13-13) showed considerable non-specific qPCR signals in neuronal cells, whereas the MACS-selected aptamer (Ast17-30) showed only a marginal qPCR signal in neuronal cells. When primary rat astrocytes and differentiated rat neurons were labeled with phycoerythrin-conjugated anti-GLAST antibody, the primary astrocytes exhibited markedly higher FL intensity (Fig. S4), further supporting the utility of anti-GLAST antibody in enriching astrocyte populations during SELEX. Specifically, Ast17-30 demonstrated a similar level of selectivity for astrocytes derived from both rat and mouse (Fig. S5). These observations indicate that MACS-assisted SELEX promoted the identification of aptamers specific to primary astrocytes. Furthermore, the use of primary astrocytes obtained from adult rat cortex and hippocampus could mitigate the developmental fluctuations in GLAST levels [29]. From the enriched aptamer pools targeting a monolayer of primary astrocytes, the MACS procedure enabled the complete separation of GLAST-positive cells (encompassing astrocytic populations) from dead cells or any co-isolated GLAST-negative cells. Furthermore, considering the *in vivo* presence of other GLAST-positive cells in the glial lineage, such as radial glial cells [30], this aptamer, which might not overlap with GLAST, can serve as an alternative ligand for the selective binding of astrocyte subpopulations. While the specific cell membrane receptors on astrocytes targeted by this aptamer were not definitively identified in this study, we postulate its interaction with specific receptors expressed by astrocytes.

### 3.2 Engineering a trivalent aptamer to enhance binding affinity to astrocytes

**Fig. 2:**
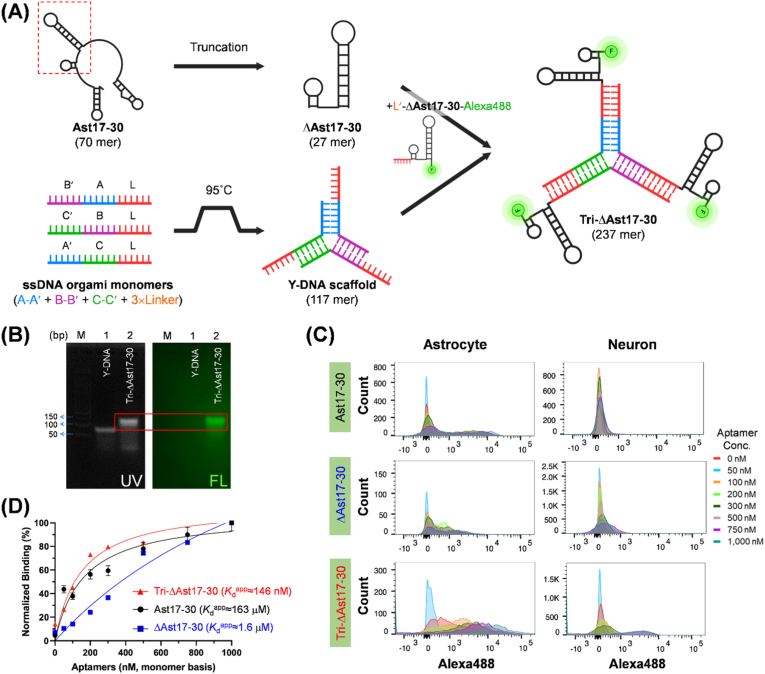
Design and characterization of truncated and trivalent aptamers derived from Ast17-30. **(A)** Schematic illustration of the design process. The original aptamer Ast17-30 was truncated to generate ΔAst17-30. Subsequently, three units of ΔAst17-30 were conjugated to a Y-DNA scaffold, followed by the addition of Alexa488 labeled ΔAst17-30 to form the trivalent aptamer Tri-ΔAst17-30. **(B)** Visualization of Tri-ΔAst17-30 by 3 % agarose gel electrophoresis under UV irradiation (left panel) and by Alexa488 FL scanning (right panel). Lane M, DNA ladder; lane 1, Y-DNA scaffold, lane 2, Tri-ΔAst17-30. The red box indicates the electrophoretic band of Alexa488-labeled Tri-ΔAst17-30 assembled through Y-DNA scaffolding. **(C)** FACS analysis of aptamer binding to primary astrocytes (left) and differentiated neurons (right). Cells were incubated with increasing concentrations (0–1000 nM) of Ast17-30, ΔAst17-30, and Tri-ΔAst17-30. The molar concentration of the trimeric aptamer was calculated as one-third of the monomeric unit (i.e., 100 nM of trimeric aptamer on the X-axis corresponds to 300 nM of monomeric aptamer). **(D)** Determination of binding affinities by quantitative FACS analysis. The normalized binding % (according to Eq. (1)) was plotted as a function of the monomer-equivalent aptamer concentration: Ast17-30 (black circles), ΔAst17-30 (blue squares), and Tri-ΔAst17-30 (red triangles). Apprarent dissociation constants (*K*_d_^app^) for each aptamer are shown in the figure. Error bars represent ±SD from three independent experiments. (For interpretation of the references to color in this figure legend, the reader is referred to the Web version of this article.)

To enhance the target binding affinity of Ast17-30 while maintaining its specificity, we engineered truncated and trivalent forms of the full-length aptamer (Fig. 2A and Table S3). Based on its predicted secondary structure, the full-length aptamer (Ast17-30, 70 mer) was truncated to a shorter variant (ΔAst17-30, 27 mer) by retaining two core hairpin structures from the original four stem-loops and removing the primer regions. Subsequently, the truncated aptamer was assembled into a Y-shaped trimeric structure (Tri-ΔAst17-30, 237 mer) using a self-assembling DNA origami scaffold technique [25,31]. Each monomer, possessing complementary motifs A/A′, B/B′, C/C′, and L, self-hybridized through complementary interactions and was assembled via controlled temperature annealing. Flanking linker (L) regions were incorporated at each arm of the Y-DNA scaffold, to which complementary linker (L′)-ΔAst17-30-Alexa488 conjugates were bound at the three termini, resulting in Tri-ΔAst17-30-Alexa488. The successful conjugation of L′-ΔAst17-30-Alexa488 to the Y-DNA scaffold was confirmed by gel electrophoresis, where the green FL signal completely co-migrated with the trimeric band (Fig. 2B). Flow cytometric analysis comparing the binding ability of Tri-ΔAst17-30 with that of the original (Ast17-30) and truncated (ΔAst17-30) aptamers revealed a significantly increased population of Alexa488-positive primary astrocytes with increasing concentrations of the trivalent aptamer (Fig. 2C). Notably, no significant change in aptamer-positive cell number for the three aptamers was observed in differentiated neurons. The FACS data also indicated a relatively higher FL intensity for the trivalent aptamer compared to the monomeric forms, even when used at monomer-equivalent concentrations (Fig. 2D). Aptamer-to-cell binding efficiency was expressed as the apparent dissociation constant (*K*_d_^app^) from FACS, which corresponds to the aptamer concentration at 50 % maximum binding in binding (%) normalized to the backgound binding in neurons, as shown in Eq. (1). As a result, Tri-ΔAst17-30 exhibited the highest binding affinity (*K*_d_^app^≈48.7 nM, trimer basis; 146 nM, monomer basis), surpassing both the full-length Ast17-30 (*K*_d_^app^≈163 nM) and the ΔAst17-30 (*K*_d_^app^≈1.6 μM). When normalized per trimeric unit, the *K*_d_^app^ of Tri-ΔAst17-30 was much lower (i.e., a stronger binding) than that of Ast17-30. Importantly, Tri-ΔAst17-30 demonstrated superior binding potency compared to the monovalent aptamers even when assessed on an equivalent ligand unit basis, strongly indicating the enhanced avidity could be driven by receptor clustering. This result further reveals that multivalent interactions between the trimeric aptamer and its receptor on astrocyte subpopulations confers a substantial avidity advantage. Taken together, these findings suggest that a Y-shaped trivalent configuration of the astrocyte-specific aptamer can enhance target-specific signal resolution owing to its increaed binding avidity.

**Fig. 3:**
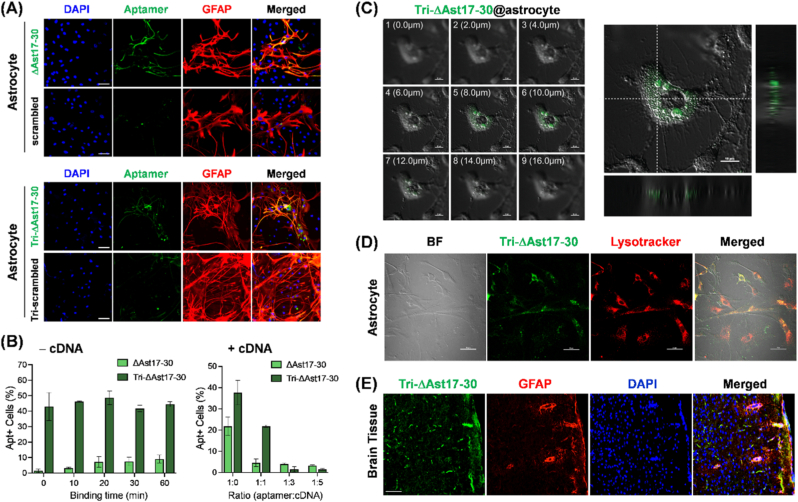
Binding and internalization of trivalent aptamers in rat astrocytes and brain tissue. **(A)** Confocal microscopy images showing the binding of Alexa488-labeled ΔAst17-30 (500 nM) and Tri-ΔAst17-30 (167 nM) to primary astrocytes (green). Cells were co-stained with DAPI (blue, nucleus) and GFAP (red). Respective scrambled aptamers labeled with Alexa488 were used as negative controls. Scale bar, 50 μm. **(B)** FACS-based aptamer cellular uptake assay in astrocytes. Time-dependent uptake of Alexa488-labeled ΔAst17-30 (green bars) and Tri-ΔAst17-30 (dark green bars) in the absence of c-DNA complementary for ΔAst17-30 (left panel). A cellular uptake inhibition assay was conducted using FACS following a 60-min co-incubation with increasing molar ratios of aptamer to c-DNA (right panel). The mixture of aptamer with c-DNA was treated with primary astrocytes for 2 h prior to FACS analysis, which quantified the percentage of aptamer-positive cells. Both Alexa488–labeled ΔAst17-30 and Tri-ΔAst17-30 was used at 500 nM; thus, the FL intensity of Tri-ΔAst17-30 is 3-fold higher than that of ΔAst17-30). The aptamer-to-cDNA ratio was calculated on the total molar amount of aptamers. Error bars represent ±SD from three independent experiments. **(C)** Z-stack confocal microscopy images showing the internalization of Alexa488-labeled Tri-ΔAst17-30 (green) by astrocytes. Optical sections were obtained at 2.0 μm intervals. The orthogonal views (right and bottom) confirm intracellular localization. Scale bar, 10 μm. **(D)** Co-localization of Alexa488-labeled Tri-ΔAst17-30 (167 nM, green) with lysotracker (red) in astrocytes, indicating lysosomal trafficking of the internalized aptamer. BF, Bright field image. Scale bar, 50 μm. **(E)** Confocal microscopy image showing the binding of Alexa488-labeled Tri-ΔAst17-30 (green) to astrocytes in rat brain tissue sections. Astrocytes were immunostained with GFAP (red), and nuclei were counterstained with DAPI (blue). Scale bar, 50 μm. (For interpretation of the references to color in this figure legend, the reader is referred to the Web version of this article.)

To further elucidate the multivalent effect of the designed aptamers, we compared astrocyte-specific imaging and uptake rates between the monovalent truncated aptamer (ΔAst17-30) and its trivalent counterpart (Tri-ΔAst17-30). Confocal microscopy confirmed the binding of both monovalent truncated (ΔAst17-30) and trivalent (Tri-ΔAst17-30) aptamers to primary astrocytes, with minimal binding observed for scrambled controls (Fig. 3A). Prolonged incubation (>2 h) of the three aptamers with astrocytes and neurons showed the strongest FL intensity for Tri-ΔAst17-30 (Fig. S6). Time-dependent uptake assays using FACS demonstrated rapid cellular internalization of the trivalent aptamer, even at the earlist time points, whereas monomeric aptamers presented relatively slower uptake over 60 min (left panel in Fig. 3B). This result indicates that Tri-ΔAst17-30 exhibits faster binding kinetics, even when considering its 3-fold higher FL intensity compared with ΔAst17-30. In addition, the relatively higher percentation of Tri-ΔAst17-30 -positive cells at binding time zero was attributed to the inherent delay between the end of the incubation and the start of FACS acquisition. To further evaluate the binding strength of the two aptamers prior to FACS analysis, we preincubated them with complementary DNA (cDNA) at varying aptamer-to-cDNA ratios, followed by cell incubation and FACS measurement (right panel in Fig. 3B). The addition of cDNA corresponding to the monomeric region inhibited aptamer internalization in a dose-dependent manner as the aptamer-to-cDNA ratio increased. However, Tri-ΔAst17-30 showed relatively delayed inhibition at ratios up to 1:3 because it could not be fully masked by cDNA at the same concentration used for ΔAst17-30. These findings highlight both the trivalency and the rapid binding capability of Tri-ΔAst17-30. Z-stack imaging confirmed the intracellular localization of Tri-ΔAst17-30 within astrocytes (Fig. 3C), and co-staining with lysotracker indicated its trafficking to lysosomes (Fig. 3D and Fig. S7). Importantly, Tri-ΔAst17-30 showed strong FL across broad regions of the rat brain tissue and partially co-localized with GFAP, a classical astrocyte marker, confirming its specific binding to astrocytes (Fig. 3E). Notably, aptamer-derived FL was also detected in GFAP-negative areas, meaning that it may recognize astrocyte subpopulations with low or heterogeneous GFAP expression. These results suggest the potential of the trivalent aptamer for effective astrocyte targeting and internalization in both *in vitro* and *ex vivo* settings. By improving cellular uptake and target engagement within tissue, the trivalent scaffold shows significant promise for achieving higher signal resolution in astrocyte-targeted applications *in vivo*. Furthermore, the observed lysosomal trafficking necessitates further research to elucidate the intracellular fate and explore potential therapeutic delivery applications of these aptamer constructs.

### 3.3 Trivalent aptamer induced gene expression changes in primary astrocyte cultures

**Fig. 4:**
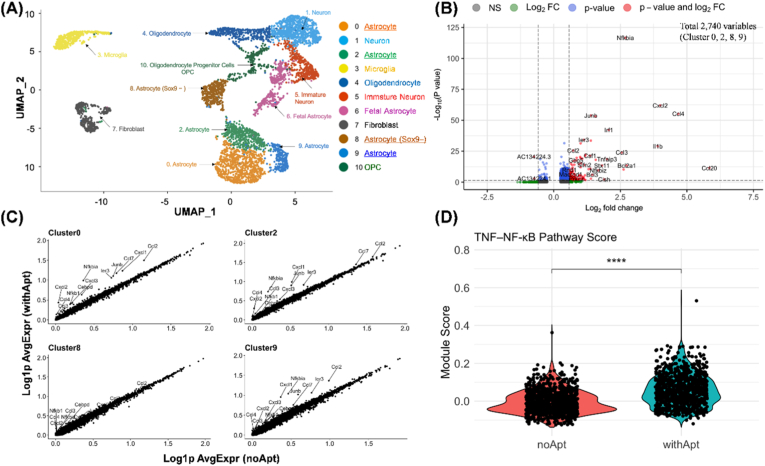
Single-cell transcriptomic analysis to observe gene expression profiles in aptamer-treated primary astrocyte cultures. **(A)** UMAP visualization of integrated scRNA seq data from primary astrocyte cultures. Individual cells are represented as dots and color-coded according to cell type clusters as indicated in the legend. The UMAP dimensionality reduction and unsupervised clustering reveals distinct cellular populations including astrocytes, neurons, oligodendrocytes, and other glial cell types within the heterogeneous culture system. **(B)** Volcano plot depicting differential gene expression analysis in astrocyte-enriched clusters (0, 2, 8, and 9) between Tri-ΔAst17-30 aptamer-treated (withApt) and untreated control (noApt) samples. The plot displays log_2_ fold change (x-axis) versus -log_10_ adjusted p-values (y-axis), with significantly upregulated genes in aptamer-treated cells highlighted in red while other tested genes were depicted in blue or green. NS, not significant (gray). **(C)** Pairwise scatter plot analysis of average gene expression (AgvExpr) between aptamer-treated and untreated cells within individual astrocyte-enriched clusters (0, 2, 8, and 9). Each plot represents log-transformed average expression level (log1p) of one gene. Genes labeled on the plots represent key inflammatory mediators and transcriptional regulators that are consistently upregulated after aptamer treatment. The diagonal line indicates equal expression between groups; genes above the line are upregulated in the aptamer-treated condition. **(D)** Violin plot of the TNF–NF-κB signaling pathway module score, defined by the average expression of canonical TNF–NF-κB signaling components. Wilcoxon rank-sum test was used to compare the two groups; the difference was statistically significant (*p* < 2.2e−16). (For interpretation of the references to color in this figure legend, the reader is referred to the Web version of this article.)

To address the impact of Tri-ΔAst17-30 aptamer treatment on primary astrocyte cultures, we performed integrated single-cell RNA sequencing (scRNA-seq) analyses on both aptamer-treated (withApt) and untreated (noApt) samples from primary rat cultured tissues. Unsupervised clustering of the scRNA-seq of the combined dataset identified multiple distinct cell populations, including astrocytes, neurons, microglia, oligodendrocytes, fibroblasts, and oligodendrocyte precursor cells (OPCs) as shown in the uniform manifold approximation and projection (UMAP) visualization (Fig. 4A). Aptamer exposure did not alter cellular states or clusters within the UMAP projection based on astrocyte marker gene expression (Fig. S8). Each cluster was annotated based on established cell-specific marker genes (Table S4) [[32], [33], [34], [35], [36], [37], [38], [39], [40], [41], [42], [43], [44], [45], [46]], revealing cellular heterogeneity within the cultured population. Importantly, four astrocyte-enriched clusters (0, 2, 8, and 9) were identified within the primary culture system, suggesting the presence of transcriptionally distinct astrocyte subpopulations. Marker gene analysis revealed differential expression of canonical astrocyte identifiers such as *Gfap*, *S100β*, and *Aqp4* across these clusters, with varying expression intensities indicative of functional specialization or divergent activation states (Fig. S9). Notably, cluster 8 emerged as a unique subpopulation characterized by the absence of Sox9 expression—a transcription factor typically associated with astrocyte maturation—implying a distinct functional role or transitional cellular state within the astrocyte lineage. Although cluster 6 was excluded from the astrocyte population because it did not express the characteristic astrocyte marker genes (*Gfap, S100β, Aqp4*), it exhibited a transcriptional signature reminiscent of fetal astrocytes, including elevated Fabp7 and reduced Aqp4 expression, suggesting the persistence of developmentally immature cells within the culture system.

To investigate aptamer-induced transcriptional changes across astrocyte subpopulations, we analyzed astrocyte-enriched clusters (0, 2, 8, and 9). Differential expression analysis between aptamer-treated and untreated samples within the merged cell population of these clusters revealed numerous genes with significantly altered expression levels (Fig. 4B and Fig. S10). Among 2740 analyzed genes (log2-FC>0.25 and min.pct>0.05), the volcano plot highlighted significant upregulation of inflammation-associated genes in aptamer-treated astrocytes. Notably, pro-inflammatory chemokines (e.g., *Cxcl2, Cxcl3, Ccl2, Ccl4, and Ccl20*) and cytokines (*Il1b* and *Tnfaip3*) were upregulated in aptamer-treated astrocytes. Scatter plots of average gene expression values confirmed consistent upregulation patterns, with key inflammatory mediators positioned above the diagonal line in each cluster. This result indicates elevated gene expression in aptamer-treated samples, with the exception of the *Sox9*-negative astrocyte cluster (Fig. 4C and Fig. S11). This uniform inflammatory signature across distinct subpopulations suggests that the aptamer targets a conserved activation mechanism, possibly via a common receptor or stress pathway rather than cluster-specific pathways. Pathway score analysis (Fig. S12) further implicated the tumor necrosis factor (TNF)-nuclear factor-kappa B (NFκB) pathway, showing statistically elevated activity post-treatment (Fig. 4D). In non-astrocyte populations, aptamer treatment modestly increased TNF–NF-κB signaling to a lesser extent than in astrocytes, likely through cytokines secreted from aptamer-stimulated astrocytes rather than direct aptamer binding (Fig. S13). Despite robust pathway activation, log_2_ fold changes for these astrocytic pro-inflammatory genes were more moderate than the magnitude of changes observed in full reprogramming to a highly reactive A1 phenotype of astrocytes [47,48], indicating a controlled inflammatory activation with a milder inflammatory stimulus. This moderate response may represent a therapeutic window for controlling astrocyte functions without triggering detrimental neuroinflammation. Consequently, these results demonstrate that Tri-ΔAst17-30 aptamer treatment induces specific and robust transcriptional responses in primary astrocytes, particularly in genes associated with inflammation and cellular activation.

### 3.4 Aptamer binding specificity in human and rat glioblastoma cell lines

**Fig. 5:**
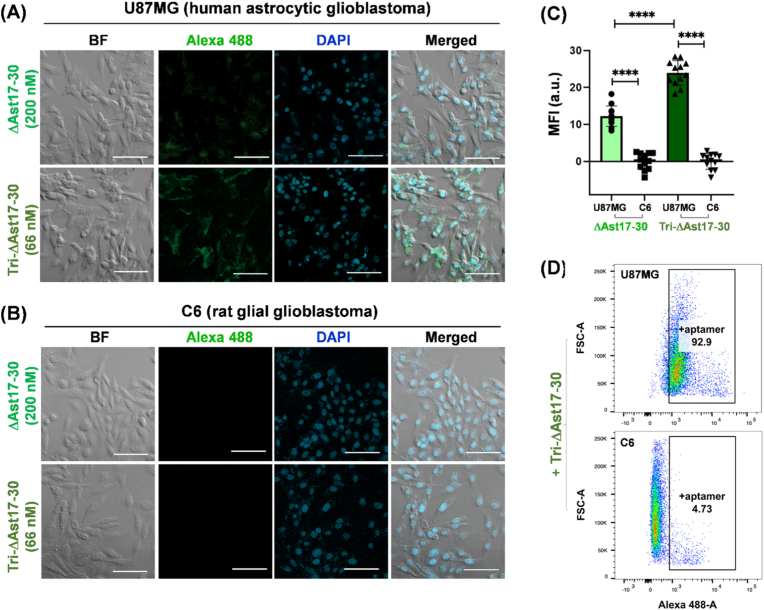
Specific binding and quantification of dye–labeled aptamers in U87MG (human astrocytic glioblastoma) and C6 (rat glial glioblastoma) cells. **(A, B)** Representative FL images of U87MG and C6 cells treated with Alexa488–labeled ΔAst17-30 (200 nM) or Tri-ΔAst17-30 (66 nM) aptamers, showing BF, Alexa488 (green), DAPI (blue), and merged channels. Scale bars, 100 μm. **(C)** Box plots of MFI values of the two aptamers showing significant differences in aptamer binding between U87MG and C6 cells (*p* < 0.0001 for both ΔAst17-30 and Tri-ΔAst17-30, *N* = 12), as well as significant difference in binding strength between the two aptamers in U87MG cells (*p* < 0.0001, *N* = 12). **(D)** Flow cytometry quantifies Alexa488–positive U87MG and C6 cells after Tri-ΔAst17-30 treatment (final 66 nM), revealing specific aptamer binding in U87MG but not C6 cells. The square gates define the percentage of aptamer-positive cells within the total population, which was 92.9 % for U87MG and 4.73 % for C6, respectively. (For interpretation of the references to color in this figure legend, the reader is referred to the Web version of this article.)

To evaluate the binding specificity and potential utility of aptamers selected by SELEX from primary rat astrocytes, we conducted binding assays in two glioblastoma cell lines with distinct origins and characteristics—U87MG, a human astrocytic glioblastoma cell line, and C6, a rat glial glioblastoma cell line. U87MG cells are widely used as a human glioblastoma model exhibiting astrocytic lineage. In contrast, C6 cells, derived from rat glioma, show different genetic and molecular profiles, including a wild-type p53 and distinct gene expression related to human glioblastoma features, but represent a more glial lineage. We treated both cell lines with Alexa 488–labeled ΔAst17-30 and Tri-ΔAst17-30 aptamers to evaluate their binding affinity and selectivity. Confocal immunofluorescence imaging demonstrated strong and specific binding of both aptamers to U87MG cells (Fig. 5A), while binding to C6 cells was minimal or negligible (Fig. 5B). This pronounced binding preference was further quantified based on FL intensity analysis of the images (Fig. 5C). In U87MG cells, Tri-ΔAst17-30 exhibited significantly higher MFI than ΔAst17-30, even when compared at monomer-equivalent concentrations. Flow cytometry analysis quantitatively confirmed this selective binding pattern, with a high percentage of Alexa 488–positive U87MG cells, whereas C6 cells showed very low FL signal (Fig. 5D). These results confirm that the Tri-ΔAst17-30 aptamer selected from primary rat astrocytes bind effectively to human astrocytic glioblastoma cells but not to the rat glial glioma line, supporting their specificity for astrocyte-like tumor cells. This finding highlights the potential of these aptamers as targeted ligands for diagnostic or therapeutic applications in astrocytic glioblastoma.

### 3.5 Trivalent anti-astrocyte aptamer enables longitudinal monitoring of astrocyte-to-iNPC conversion

**Fig. 6:**
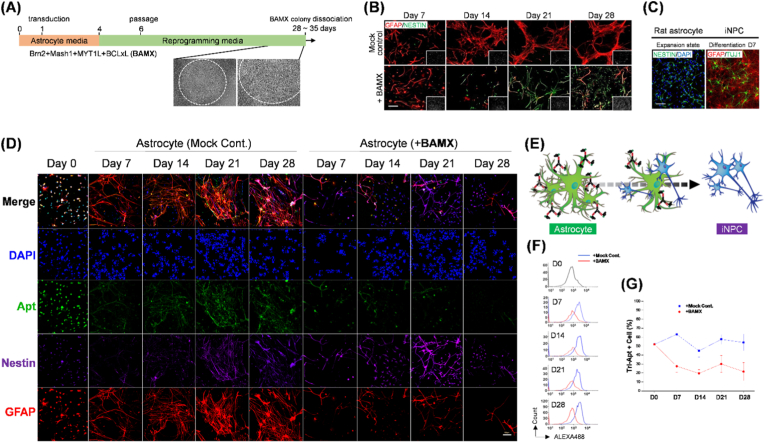
Longitudinal monitoring of the direct conversion of astrocytes into induced neural progenitor cells (iNPCs) using Tri-ΔApt17-30. **(A)** Schematic timeline of the direct reprogramming process. Rat astrocytes were transduced with BAMX factors (*Brn2*, *Ascl1*/*Mash1*, *Myt1L*, and *Bcl-xL*) and cultured in astrocyte media for 4 days, and then in reprogramming media. BAMX colony dissociation was performed between 28 and 35 days. Representative phase-contrast images of the cell morphology during reprogramming are shown on the right. **(B)** Immunocytochemistry analysis of astrocyte marker (GFAP, red) and neural progenitor cell marker (Nestin, green) at different time points (Day 7, 14, 21, and 28) in mock-control and BAMX-transduced astrocytes. Insets in the bottom right of each image show the corresponding brightfield views. Scale bar, 100 μm. **(C)** Immunocytochemistry of rat astrocyte-derived iNPCs at the expansion state (Nestin, green; DAPI, blue) and after seven days of differentiation (GFAP, red; Tuj1, green). Scale bar, 100 μm. **(D)** Time-course confocal images showing the staining of DAPI (blue, neclei), the binding of Tri-ΔApt17-30 (green, detected via Alexa488), and the expressions of Nestin (purple, intracellular marker for neuron) and GFAP (red, intracellular marker for astrocyte) in mock-control astrocytes and BAMX-transduced astrocytes at different time points (Day 0, 7, 14, 21, and 28) during the reprogramming process. Scale bar, 100 μm. **(E)** Schematic illustration depicting the binding of the Alexa488-conjugated aptamer Tri-ΔApt17-30 (red) to the surface of astrocytes and the subsequent morphological changes as they are reprogrammed into iNPCs. **(F)** Flow cytometry histograms showing the binding of Alexa488-Tri-ΔApt17-30 to cells at different time points (D0, D7, D14, D21, D28) in mock-control (+Mock Cont., blue line) and BAMX-transduced (+BAMX, red line) cultures. **(G)** Quantification of the percentage of cells positive for Tri-ΔApt17-30 binding over time in mock-control (blue squares) and BAMX-transduced (red circles) cultures. Error bars represent ±SD from three independent experiments. (For interpretation of the references to color in this figure legend, the reader is referred to the Web version of this article.)

To elucidate the direct reprogramming of rat primary astrocytes into induced neural progenitor cells (iNPCs) mediated by BAMX factors, we conducted a longitudinal analysis integrating molecular marker expression profiles with aptamer-based cell surface monitoring. The successful induction of iNPCs was illustrated in Fig. 6A−6C. Over a period of 28–35 days post-transduction, BAMX-treated cultures exhibited morphological alterations consistent with a neural progenitor cell fate (Fig. 6A). IHC analysis revealed a temporal transition, characterized by a reduction in the astrocyte marker GFAP and a concomitant increase in the neural progenitor marker Nestin in BAMX-treated cells, particularly evident at Days 21 and 28 (Fig. 6B). This contrasted sharply with the mock-control group, which largely retained GFAP expression. Furthermore, the generated iNPCs at the expansion stage expressed Nestin, and upon differentiation, displayed markers characteristic of both astrocytes (GFAP) and neurons (TUJ1) (Fig. 6C). These observations were corroborated by RT-qPCR and its quantitative analysis (Fig. S14), which demonstrated an upregulation of neural progenitor markers (*Nestin* and *Sox2*) and a downregulation of the astrocyte marker (*Gfap*) throughout the reprogramming time course in BAMX-transduced cells, mirroring the expression patterns observed in control rat neural progenitor cells (rNPCs).

To ascertain whether the astrocyte-specific aptamer could monitor cell surface dynamics during this cellular transition, we employed Alexa488-conjugated Tri-ΔApt17-30. The conceptual design of this monitoring strategy is presented in Fig. 6D. Time-lapse imaging revealed a progressive decline in aptamer binding (green) in BAMX-transduced astrocytes, temporally correlated with morphological and molecular marker changes indicative of iNPC conversion. In contrast, the mock-control group exhibited sustained aptamer binding signals. Quantitative flow cytometry analysis (Fig. 6E, 6F, and 6G) further confirmed this trend, showing a significant reduction in aptamer-positive cells within the BAMX-treated population over time, whereas aptamer binding remained stable in the mock-control group. These findings demonstrate dynamic changes in astrocyte-specific aptamer binding and highlight the efficacy of BAMX factors in directly converting rat astrocytes into iNPCs, as evidenced by the temporal modulation of key molecular markers. Importantly, the reduced Tri-ΔApt17-30 binding in the BAMX-treated cells likely reflects downregulation or structural reorganization of astrocyte-specific surface markers during iNPC conversion. This indicates that the aptamer treatment did not influence astrocyte reprogramming. Consequently, pro-inflammatory gene induction by aptamers (Fig. 4) possibly represents a reporter effect rather than the primary conversion mechanism. Although the specific surface proteins on reprogrammed astrocytes were not elucidated in this study, this aptamer-based approach provides a valuable tool for real-time tracking of cell surface remodeling during cellular transitions.

Collectively, our findings demonstrate that the trivalent aptamer (Tri-ΔApt17-30), with its specific affinity for the astrocyte surface, offers significant advantages over conventional antibody-based methods for monitoring cellular reprogramming. Unlike traditional cytoplasmic markers (e.g., α-GFAP) and even the existing surface marker antibody (i.e., α-ACSA-1), which necessitate cell fixation and permeabilization and are thus incompatible with live-cell tracking, surface-binding aptamers enabled non-invasive, real-time monitoring of living cells. This crucial feature facilitated longitudinal studies of individual cells undergoing dynamic identity changes, which is essential for understanding the heterogeneity and temporal dynamics of the reprogramming process at the single-cell level. Furthemore, aptamers can be readily engineered or chemically modified to permit straightforward customization for diverse applications, including imaging, cell sorting, and targeted drug delivery. In contrast to fluorescent protein reporters driven by lineage-specific promoters [49] or optogenetic cacium modulation [50], which demand genetic modification and consequently limit their use in primary cells or tissues, aptamers offer a complelling flexibility by targeting native surface epitopes across heterogenous astrocyte subpopulations with minimal pertumation to cellular physiology. Compared to optical diffraction tomography, which enables real-time monitoring of astrocyte morphological dynamics [51], our aptamer approach provides direct identification of astrocytes through the cell surface, allowing precise mapping and analysis of distinct cell populations. Nevertheless, translating this method for *in vivo* applications presents significant hurdles, including overcoming the blood-brain barrier and preventing aptamer degradation by nucleases. Therefore, a more immediately feasible clinical application may involve direct local administration of the aptamer to target tissues after surgical intervention. As a foundational step toward therapeutic use, we expanded our investigation to a disease model, confirming that our aptamer effectively binds to astrocytic glioblastoma cells (Fig. 5). Additionally, we observed that the aptamer treament primarily induced moderate activation in mature astrocytes, and this effect was unrelated to differentiation. This is supported by the minimal pro-inflammatory gene expression in *Sox9*-negative astrocytes (Fig. 4C) and the decreased aptamer binding during astrocytes transdifferentiation (Fig. 6). Importantly, no significant increase in intracellular ROS levels was observed when glioblastoma cells were treated with the aptamer (Fig. S15). Consequently, the TNF–NF-κB-mediated responses observed in aptamer-treated astrocytes (Fig. 4D) possibly represent a normal adaptive response rather than a detrimental inflammatory process. Although the specific astrocyte surface receptors recognized by this aptamer could not be identified in this study due to technical limitations, our RNA-seq analysis revealed increased *Cd14* gene expression within the astrocyte receptor gene profile, which may be associated with ssDNA-induced activation of the TNF–NF-κB pathway (Fig. S16). Because CD14 functions as a co-receptor for TLR family and other pattern recognition receptors, it could contribute to aptamer recognition and downstream signaling. It is also plausible that CD14 facilitates the internalization of nucleic acid ligands through a multivalent clustering process [52,53]. Future research will focus on characterizing aptamer binding targets across astrocyte subtypes. Given the increasing recognition of astrocyte functional diversity [54,55], this aptamer-based strategy holds considerable promise for bridging gaps in our ability to observe both the temporal and spatial dynamics of astrocyte biology, providing a robust and versatile tool for studying transdifferentiation and physiological functions.

## 4 Conclusions

In conclusion, we developed a novel trivalent DNA aptamer (Tri-ΔAst17-30) that selectively binds to the astrocyte cell surface with increased binding avidity. This enabled longitudinal tracking of the same cell population and their conversion into iNPCs. This aptamer-based strategy offers distinct advantages over conventional destructive assays or antibody-based imaging by facilitating minimally invasive, time-lapse monitoring of live astrocytes without the need for viral reporters and with negligible impact on differentiation. Furthermore, Tri-ΔAst17-30 aptamer exhibited specificity for astrocyte-like tumor cells. Consequently, this approach holds substantial promise for unraveling the dynamic and heterogeneous processes of astrocyte transdifferentiation and for elucidating their diverse physiological roles over time. We anticipate that extending our aptamer-based approach to other cell lineages will greatly enhance the broad applicability and influence of this imaging platform.

## CRediT authorship contribution statement

**Bohyun Oh:** Investigation, Formal analysis, Data curation, Validation, Visualization, Writing – review & editing. **Eun-Song Lee:** Writing – original draft, Visualization, Validation, Investigation, Data curation. **Eun-Hye Lee:** Visualization, Validation, Investigation, Data curation. **Kyung-Min Kim:** Investigation, Data curation. **Yeonju Lee:** Investigation, Data curation. **Jin-Sam Lee:** Investigation. **Hong-Gyun Lee:** Supervision. **Hyobin Jeong:** Writing – review & editing, Supervision, Data curation. **Chang-Hwan Park:** Writing – review & editing, Writing – original draft, Supervision. **Young-Pil Kim:** Writing – review & editing, Writing – original draft, Supervision, Funding acquisition.

## Declaration of competing interest

The authors declare that they have no known competing financial interests or personal relationships that could have appeared to influence the work reported in this paper.

## Data Availability

Data will be made available on request.
