# Supplementary material for: Y-shaped trivalent aptamer for targeted visualization and tracking of reprogrammed astrocytes
Source: Mater Today Bio. 2025 Oct 28;35:102482. doi: 10.1016/j.mtbio.2025.102482 (PMC12621448; doi:10.1016/j.mtbio.2025.102482)
Supplement: Multimedia component 1 [file mmc1.docx]

**Supplementary Data**

**Y-shaped trivalent aptamer for targeted visualization and tracking of reprogrammed astrocytes**

Bohyun Oh^a,†^, Eun-Song Lee^a,†^, Eun-Hye Lee^b,c†^, Kyung-Min Kim^a^, Yeonju Lee^a^, Jin-Sam Lee^d^, Hong-Gyun Lee^e^, Hyobin Jeong^f,^*, Chang-Hwan Park^d,g,^**, and Young-Pil Kim^a,h,i,^***

^a^Department of Life Science, Hanyang University, Seoul 04763, Republic of Korea

^b^Neuroregeneration and Stem Cell Programs, Institute for Cell Engineering, Johns Hopkins University School of Medicine, Baltimore, MD 21205, USA

^c^Department of Neurology, Johns Hopkins University School of Medicine, Baltimore, MD 21205, USA

^d^Hanyang Biomedical Research Institute, Hanyang University, Seoul 04764, Republic of Korea

^e^School of Biological Sciences, Seoul National University, Seoul 08826, Republic of Korea

^f^Department of Systems Biology, College of Life Science and Biotechnology, Yonsei University, Seoul 03722, Republic of Korea

^g^Bureau of Research & Development Innovation, Korea Health Industry Development Institute, Cheongju 28159, Republic of Korea

^h^Research Institute for Convergence of Basic Science & Research Institute for Natural Sciences, Hanyang University, Seoul 04763, Republic of Korea

^i^Hanyang Institute of Bioscience and Biotechnology, Hanyang University, Seoul 04763, Republic of Korea

**
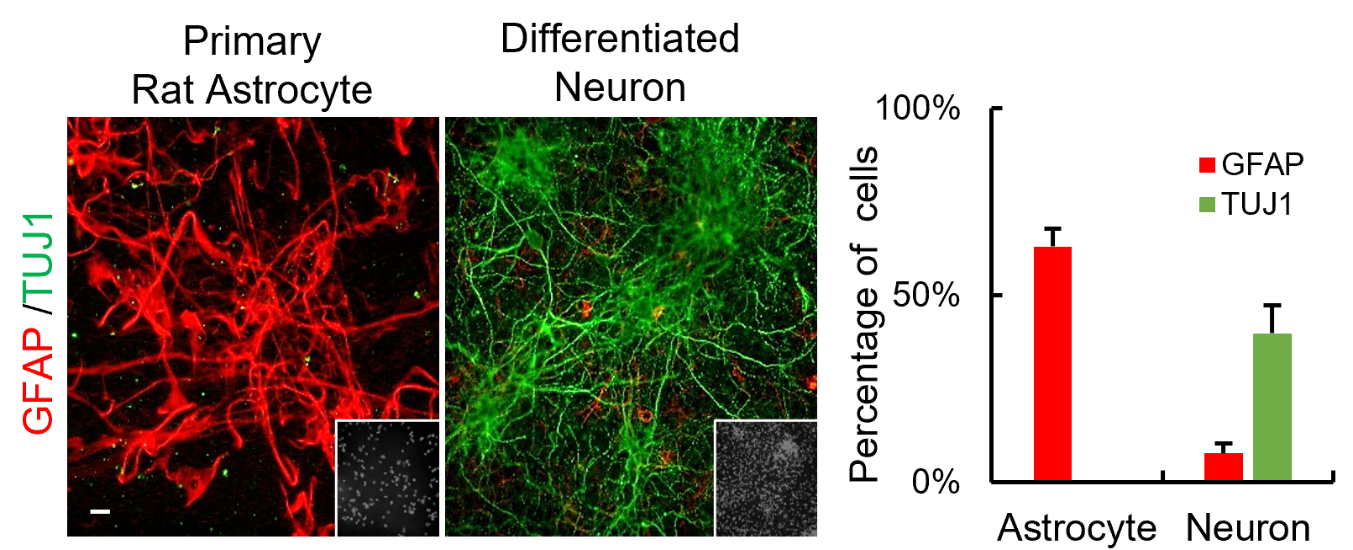
**

**Fig. S1**. Immunocytochemical characterization of primary rat astrocytes and differentiated neurons employed in cell-SELEX. Confocal microscopy reveals GFAP (red) labeling astrocytes and TUJ1 (green)-labeled neurons. A predominantly astrocyte culture (left image) and a neuron-differentiated culture (right image) are shown, with corresponding brightfield insets (bottom right). Scale bar, 20 µm. The accompanying bar graph quantifies the percentage of GFAP-positive (red) and TUJ1-positive (green) cells in each culture, illustrating the high purity of the astrocyte preparation and the neuronal enrichment of the mixed population. Error bars represent ±SD from three independent experiments.


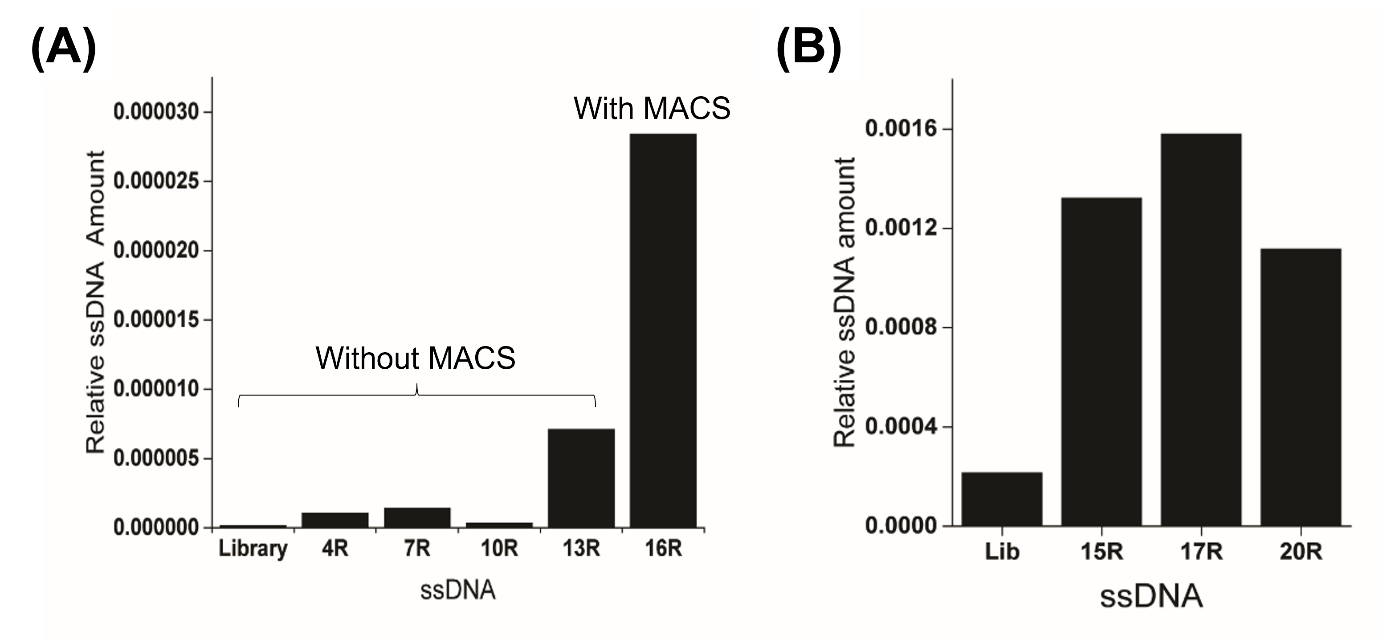


**Fig. S2**. Real-time qPCR-based quantification of the ssDNA library binding to primary astrocytes during SELEX rounds. **(A)** Comparative quantification of the ssDNA library binding in rounds without MACS (rounds 4, 7, 10, and 13) versus a round with MACS (round 16). **(B)** Comparative quantification of ssDNA library binding across rounds 15, 17, and 20 during MACS-assisted SELEX. The highest ssDNA binding capacity was observed in round 17.


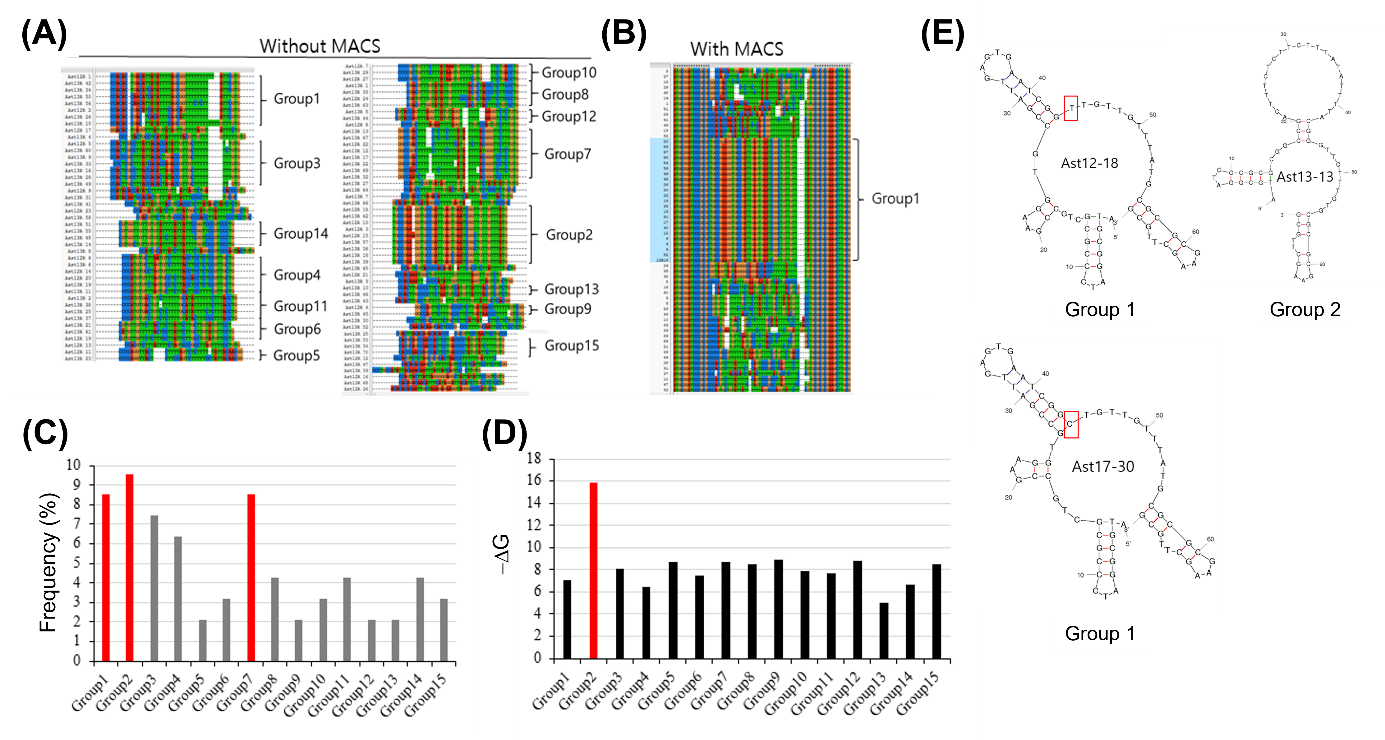


**Fig. S3**. Selection and analysis of astrocyte-specific aptamer candidates. **(A** and **B)** Multiple sequence alignment of selected aptamers enriched after cell-SELEX without (1 to 13 rounds) and with MACS (14 to 20 rounds). **(C)** Frequency distribution of aptamer candidates showing the relative abundance of each sequence group from (A) and (B). The three highest frequencies were observed in groups 1, 2, and 7 (red bar). **(D)** Thermodynamic stability (ΔG) analysis of each aptamer group. The highest thermodynamic stability was observed in group 2 (red bar). **(E)** Predicted secondary structures of astrocyte-binding aptamer candidates: Ast12-18 (group 1), Ast13-13 (group 2), and Ast17-30 (group 1). Ast12-18 and Ast17-30 have the same sequence except for one nucleotide (red box), but their secondary structures differ in a small bulge region.


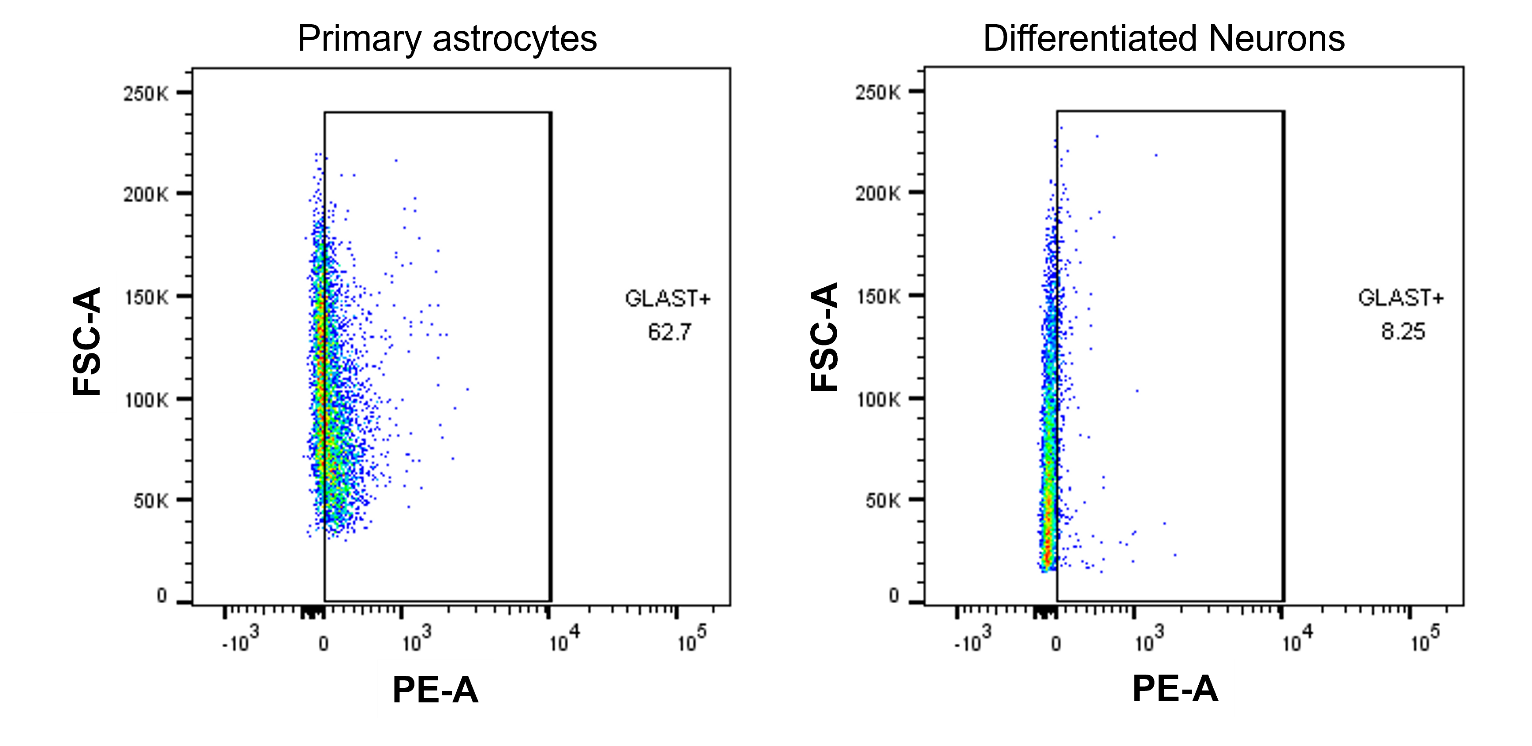


**Fig. S4**. Flow cytometric analysis of primary rat astrocytes and differentiated rat neurons labeled with phycoerythrin (PE)-conjugated anti-GLAST antibody. Left panel: Primary astrocytes exhibited a high proportion of GLAST-positive cells (62.7%), as indicated by increased PE-A FL. Right panel: Differentiated neurons show minimal GLAST expression, with only 8.25% GLAST-positive cells detected. FSC-A, forward scatter area; PE-A, phycoerythrin area. These results demonstrate the specificity of the anti-GLAST antibody for astrocyte populations among primary cells.


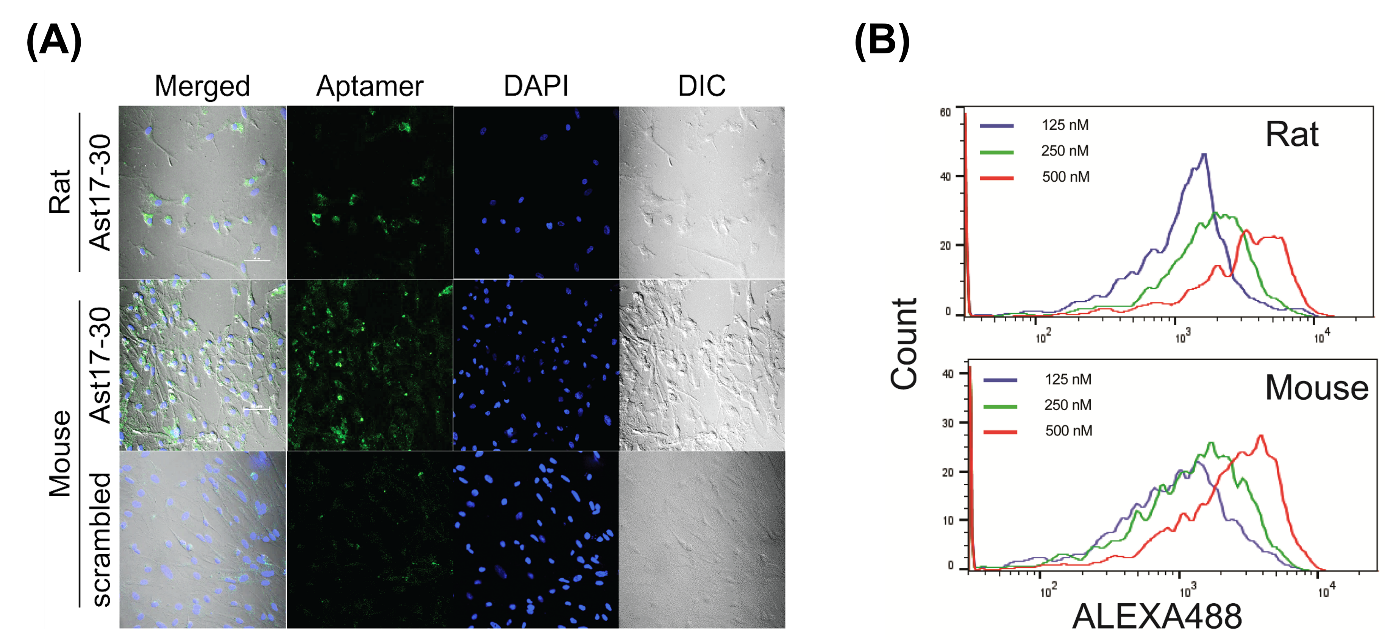


**Fig. S5**. Specific binding of Ast17-30 to rat and mouse astrocytes. **(A)** Confocal microscopy images demonstrating the binding specificity of the Alexa488-conjugated Ast17-30 aptamer to astrocytes. Rat primary astrocytes (top row) and mouse primary astrocytes (middle row) were incubated with the Alexa488-Ast17-30 aptamer. A scrambled aptamer conjugated with Alexa488 was used as a negative control on mouse primary astrocytes (bottom row). The columns represent merged images (Alexa488, DAPI, and DIC), Alexa488 fluorescence (green, indicating aptamer binding), DAPI staining (blue, indicating cell nuclei), and differential interference contrast (DIC) images showing cell morphology. Scale bar, 50 µm. **(B)** Flow cytometry quantifying the binding of the Alexa488-Ast17-30 aptamer to rat (top panel) and mouse (bottom panel) primary astrocytes at different concentrations (125 nM, 250 nM, and 500 nM). The histograms show the cell count (*y*-axis) versus the Alexa488 FL intensity (*x*-axis), indicating the level of aptamer binding.


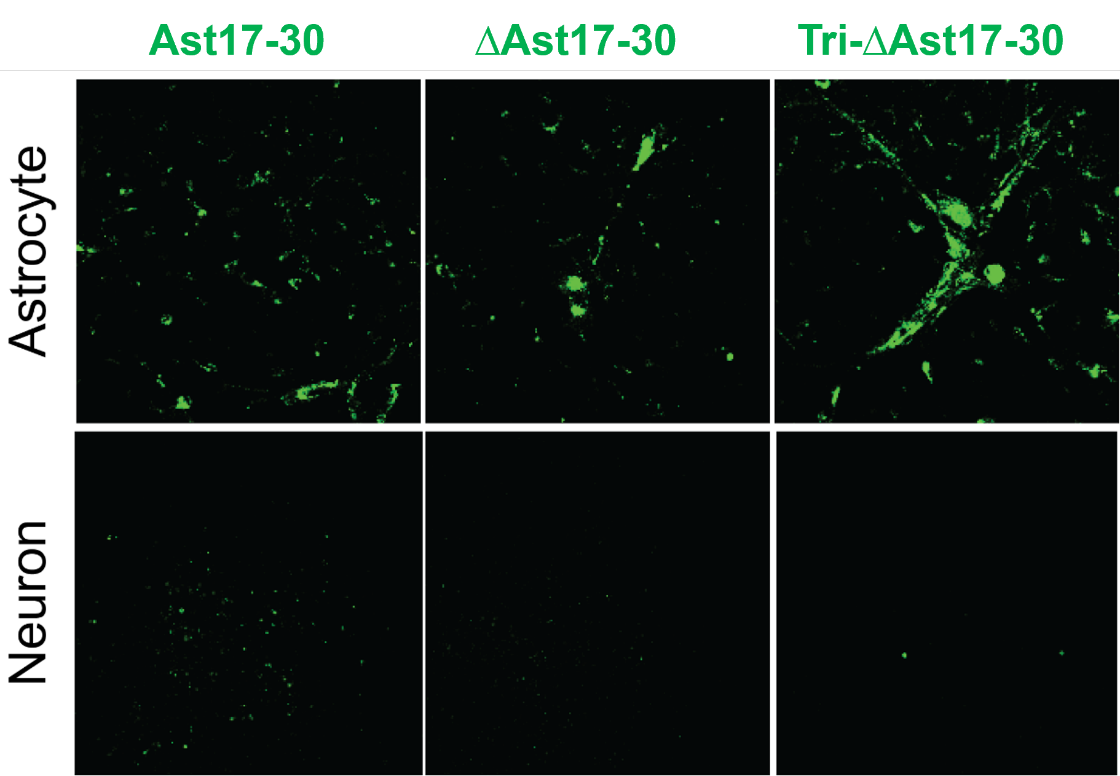


**Fig. S6**. **Confocal microscopy images showing the binding of Alexa488-conjugated aptamers (Ast17-30, tAst17-30, and Tri-tAst17-30) to primary astrocytes (top row) and neurons (bottom row) after a 2-h incubation.** The green FL indicates aptamer binding.


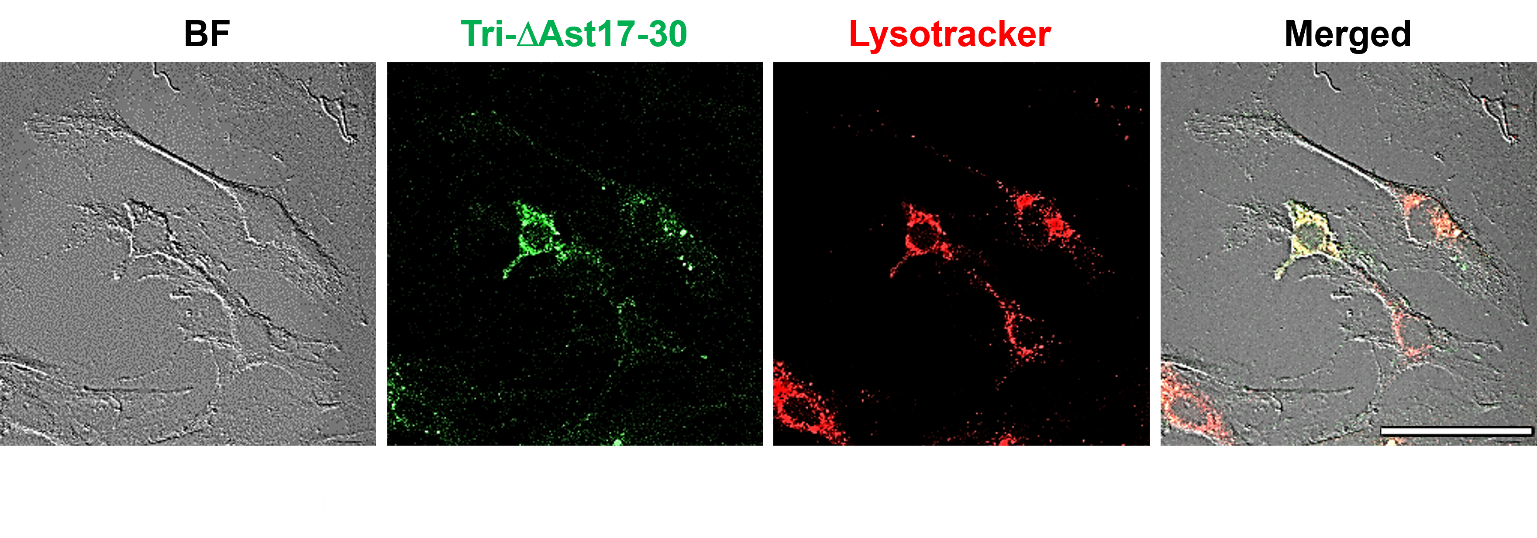


**Fig. S7.** High-magnification image corresponding to Fig. 3D. The image shows the co-localization of Alexa488-labeled Tri-ΔAst17-30 (green) with Lysotracker (red) in rat astrocytes. Scale bar, 50 µm.


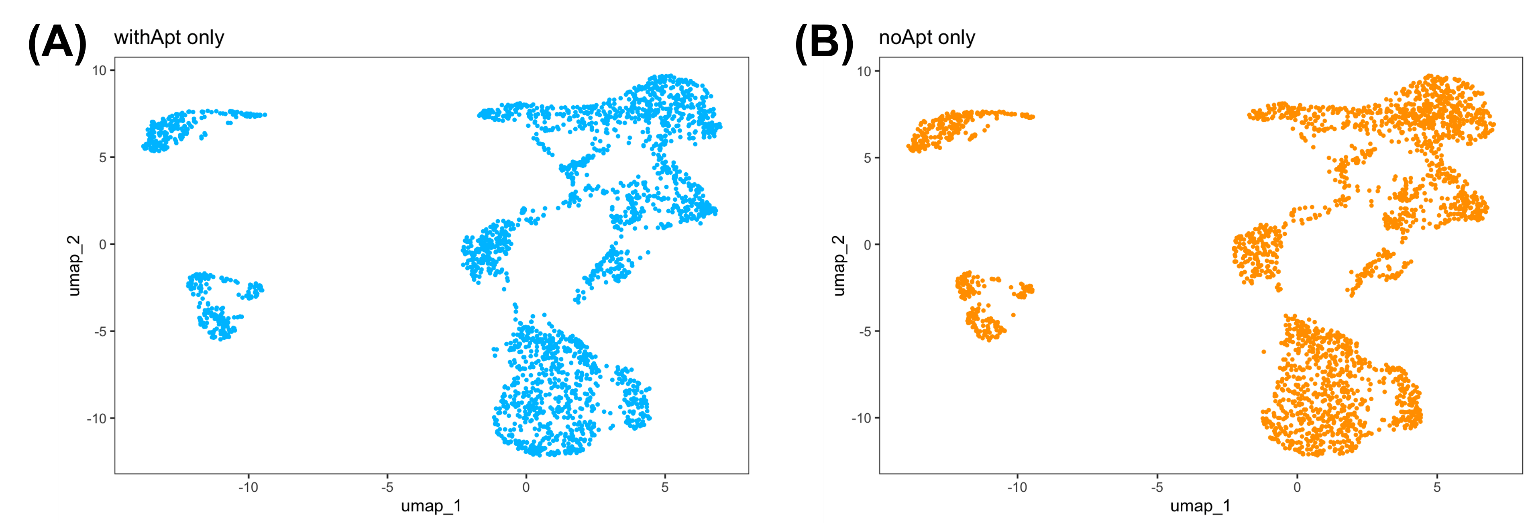


**Fig. S8.** UMAP visualization of primary cells based on astrocyte marker gene expression. (A) Cells treated with aptamer (withApt, blue) and (B) untreated controls (noApt, orange) are projected onto the same dimensionality reduction plot. Gene expression–based clustering showed no significant difference between the two populations.


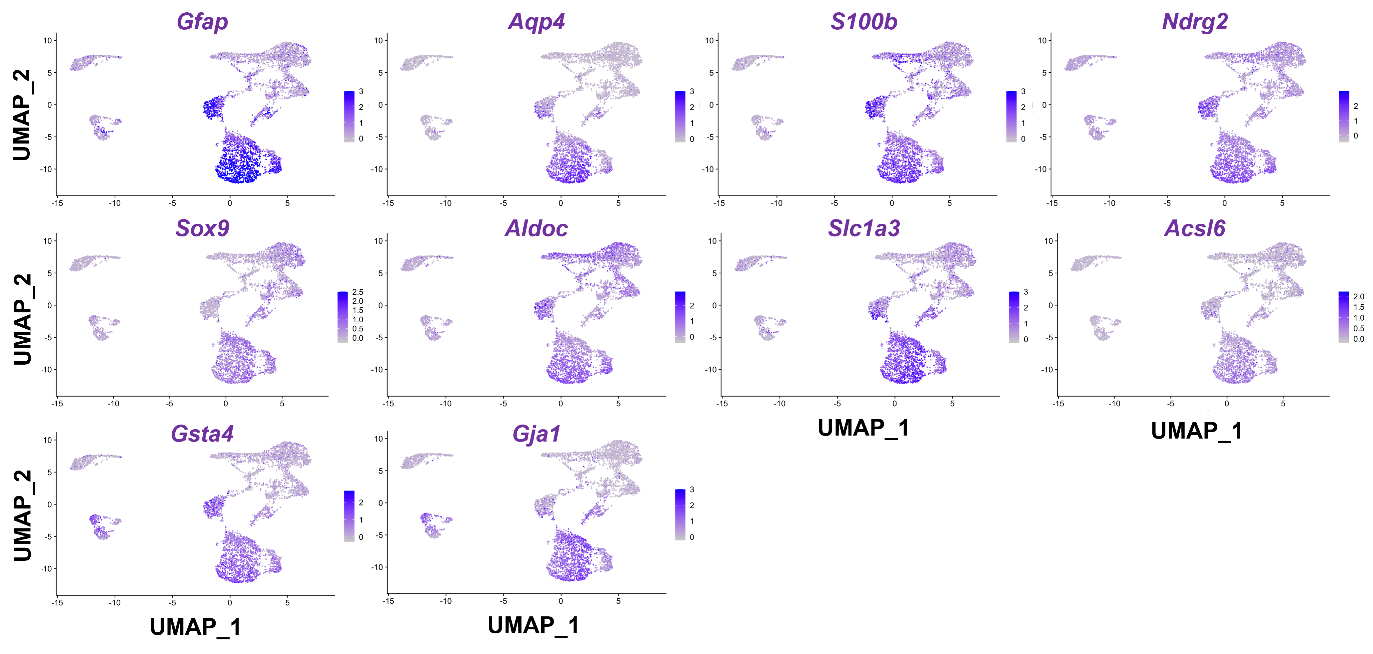


**Fig. S9.** Expression patterns of astrocyte marker genes in scRNA-seq clusters, as visualized by UMAP in **Figure 4A**. Each panel displays the expression of a specific astrocyte marker gene (*Gfap, Aqp4, S100b, Ndrg2, Sox9, Aldoc, Slc1a3, Acsl6, Gsta4,* and *Gja1*) across all cells, projected onto a two-dimensional UMAP plot. Each dot represents an individual cell, with color intensity reflecting the normalized expression level of the indicated gene; darker blue denotes higher expression. This visualization illustrates the spatial distribution of astrocyte marker gene expression across the identified cell clusters.


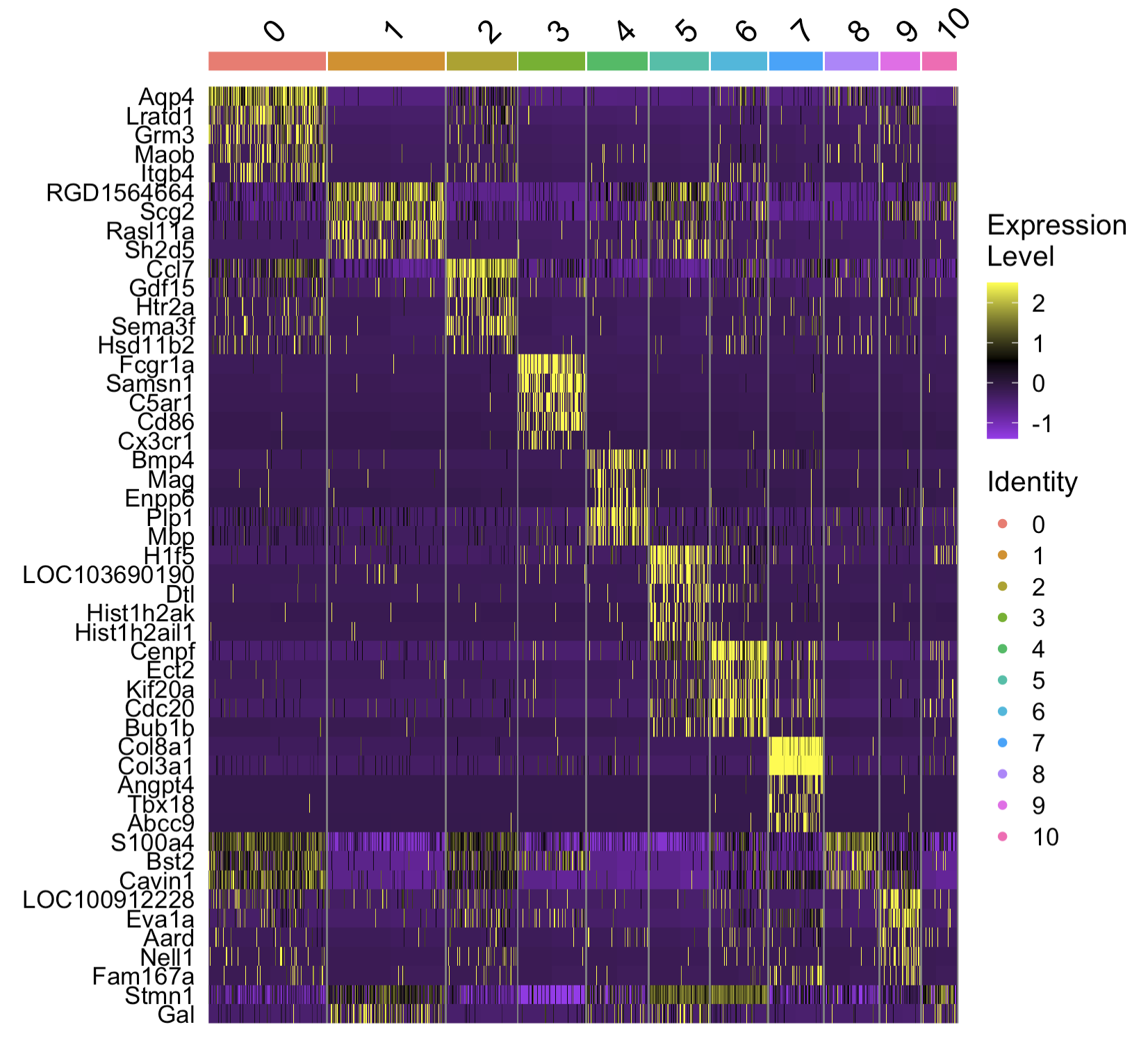


**Fig. S10**. Heatmap of the top five variable genes across identified cell clusters. The heatmap displays the top five most variable genes for each of the 11 clusters (0-10) identified from integrated single-cell RNA sequencing of primary rat astrocytes treated with or without Tri-∆Ast17-30 aptamer. Clusters were determined following data integration and dimensionality reduction analysis. Expression levels are shown on a color scale from purple (low expression, 0) to yellow (high expression, 2). The distinct expression patterns reveal cluster-specific transcriptional signatures that distinguish astrocyte subpopulations and non-astrocyte cell types within the dataset.


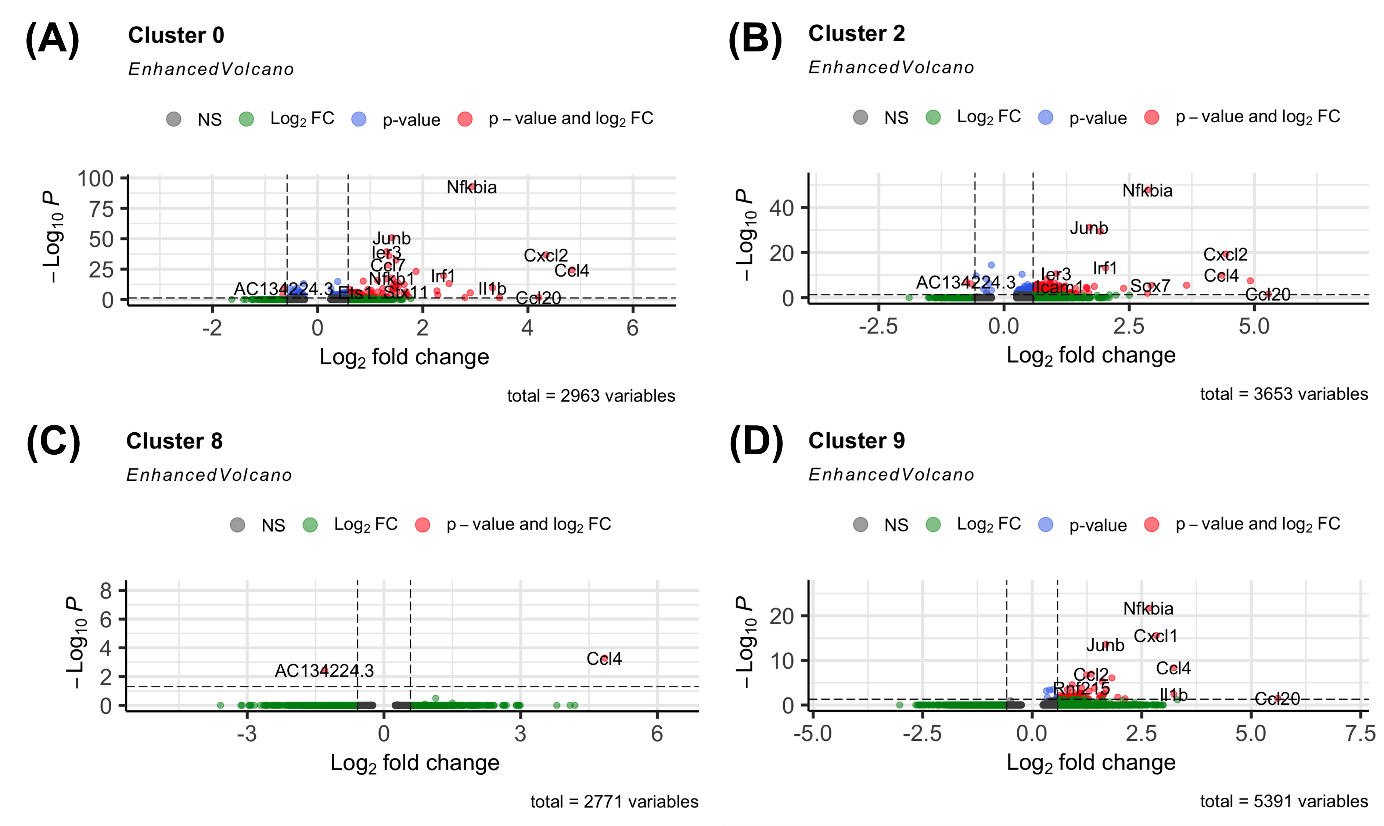


**Fig. S11**. Volcano plot depicting differential gene expression analysis in each astrocyte-enriched cluster (0, 2, 8, and 9, corresponding A, B, C, and D) between Tri-ΔAst17-30 aptamer-treated (withApt) and untreated control (noApt) samples. The plot displays log_2_ fold change (x-axis) versus -log_10_ adjusted p-values (y-axis), with significantly upregulated genes in aptamer-treated cells highlighted in red while other tested genes were depicted in blue or green. NS, not significant (gray).


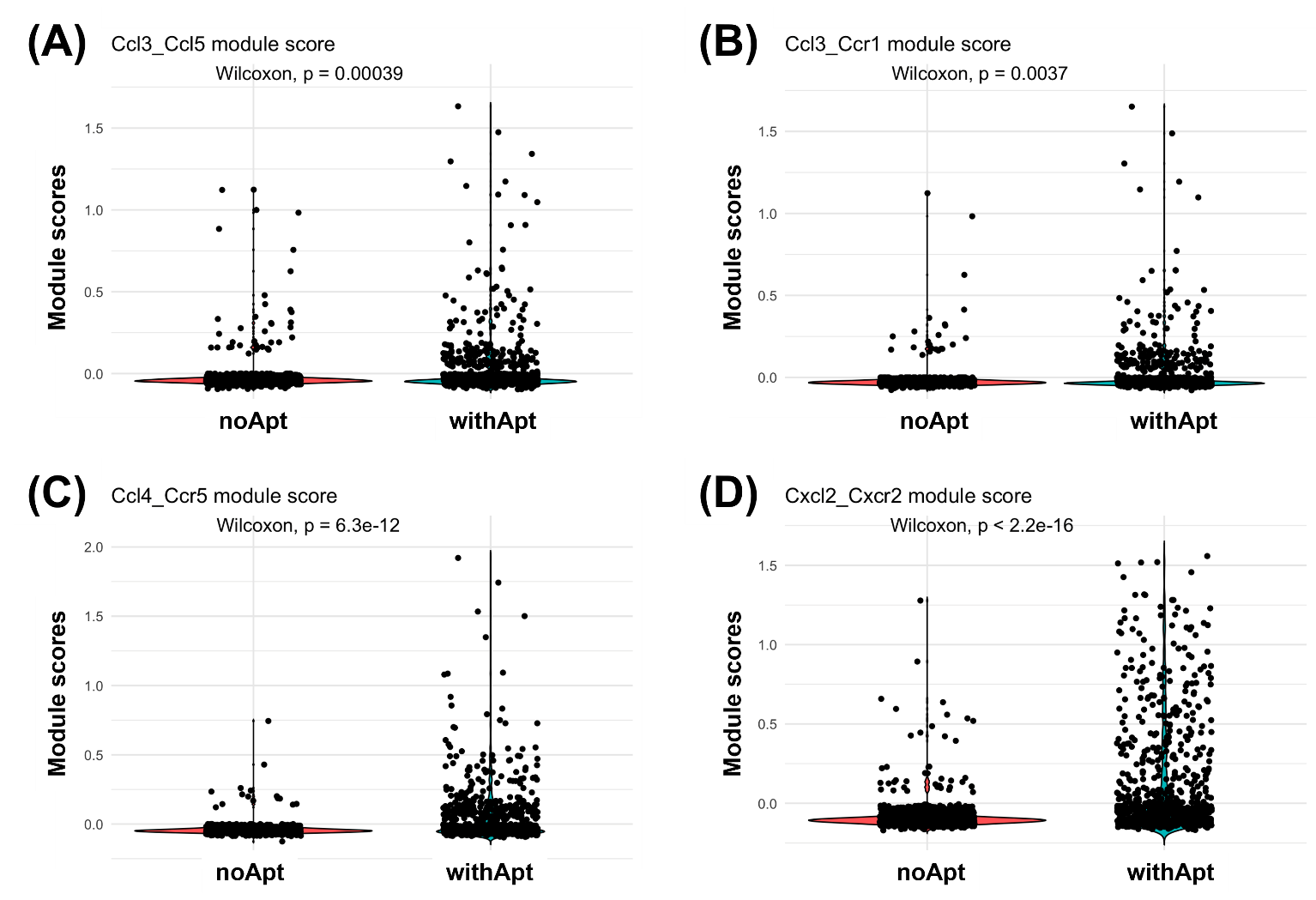


**Fig. S12**. Module scores for cytokine signaling axes in astrocyte-enriched clusters. Violin plots comparing module scores for key cytokine-ligand receptor signaling pathways between untreated (noApt, red) and aptamer-treated (withApt, blue) conditions in astrocyte-enriched clusters (0, 2, 8, 9). Module scores were calculated based on KEGG pathway annotations for *Rattus norvegicus* to assess cytokine-mediated immune cell activation through ligand-receptor interactions: (A) *Ccl3-Ccl5* axis, (B) *Ccl3-Ccr1* axis, (C) *Ccl4-Ccr5* axis, and (D) *Cxcl2-Cxcr2* axis. Statistical significance was determined using Wilcoxon rank-sum tests. While significant differences were observed across all four cytokine signaling axes, the magnitude of change was moderate rather than dramatic. The *Cxcl2-Cxcr2* axis showed the most substantial elevation in module scores following aptamer treatment, suggesting selective enhancement of specific cytokine-receptor interactions rather than broad immune activation. The moderate increases in cytokine-receptor module scores likely reflect the downstream consequence of TNF-NFκB-driven transcription of chemokines like *Cxcl2* and *Ccl4*. This suggests a controlled inflammatory activation where gene transcription is robustly upregulated through TNF-NFκB, but actual cytokine-mediated immune cell recruitment remains measured.


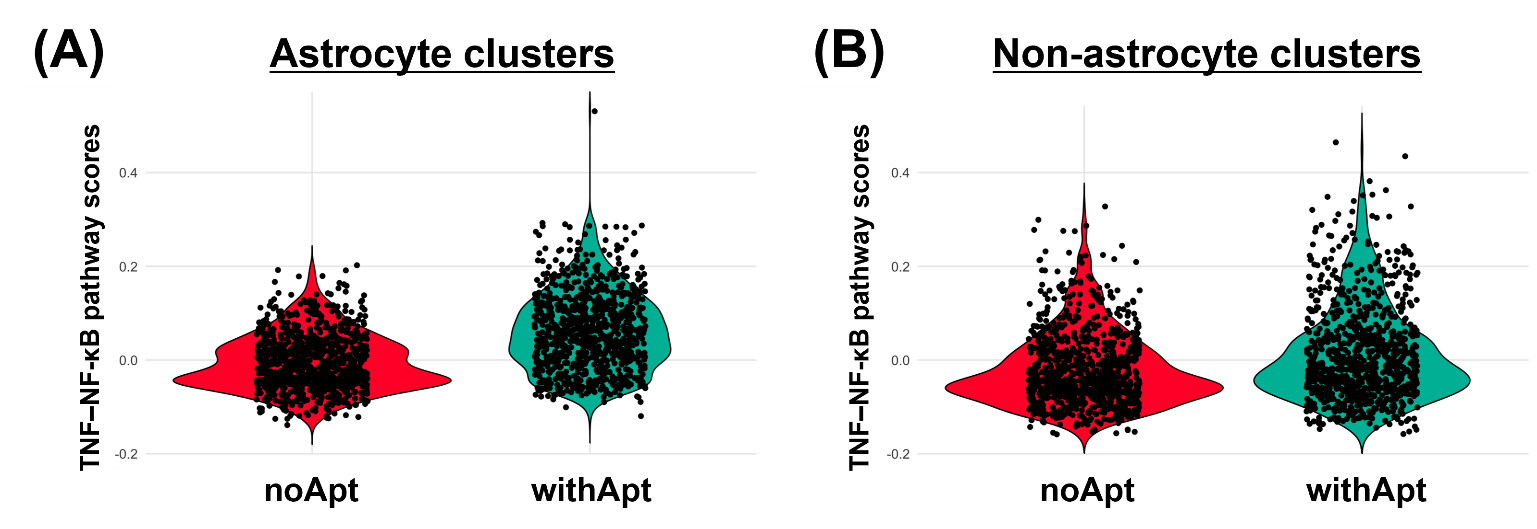


**Fig. S13**. Comparison of violin plots of the TNF–NF-κB pathway scores between astrocyte-enriched clusters (0, 2, 8, and 9) **(A,** identical to **Fig. 4D**) and non-astrocyte-enriched clusters (1, 3, 4, 5, 6, 7, and 10) **(B)** in the absence and presence of Tri-ΔAst17-30 aptamer, defined by the average expression of canonical TNF–NF-κB signaling components. The Wilcoxon rank-sum test was used to compare the noApt and withApt groups. A more significant difference was observed for astrocytes (*n*=988 *vs*. 950, median=−0.0135 *vs.* 0.0490, *p*=1.73×10⁻^64^, AUC=0.235, Cliff’s δ = −0.530) than that for non-astrocytes (*n*=1,557 *vs*. 1,540, median=−0.0398 *vs*. −0.0076, *p*=2.90×10⁻^13^, AUC=0.106, Cliff’s δ = −0.788).


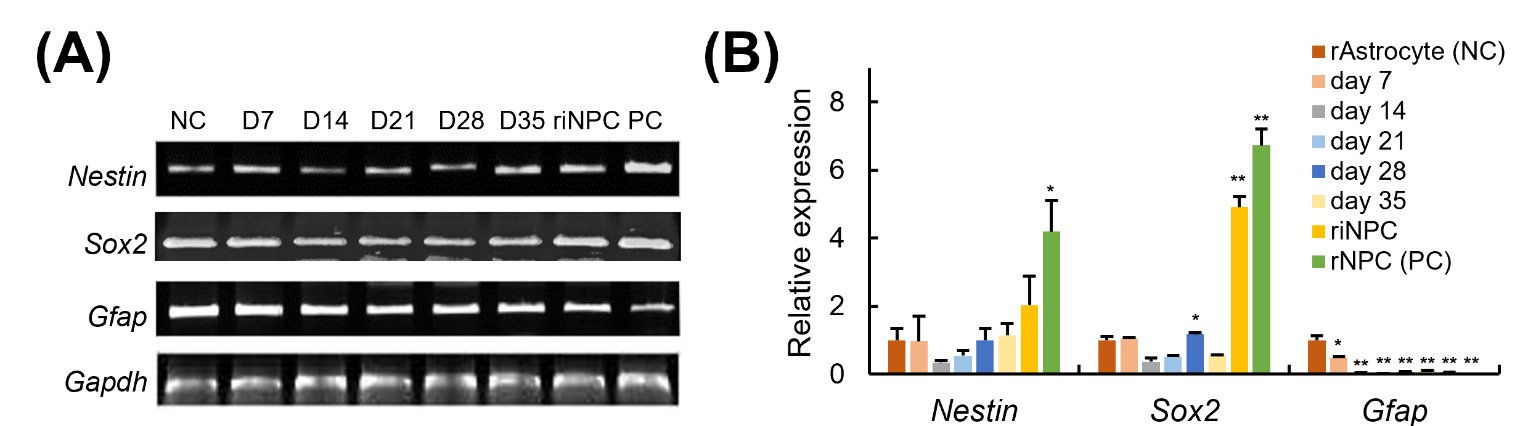


**Fig. S14**. Changes in gene expression associated with BAMX factor-mediated direct conversion of rat astrocytes (rAstrocyte) into induced neural progenitor cells (riNPC). **(A)** RT-qPCR analysis of the expression of NPC markers (*Nestin* and *Sox2*), an astrocyte marker (*Gfap*), and a housekeeping gene (*Gapdh*) at different time points during reprogramming (NC: negative control, D7, D14, D21, D28, D35) and in rat neural progenitor cells (rNPC) as a positive control (PC). **(B)** Quantification of the relative gene expression levels from RT-qPCR data, normalized to *Gapdh*. Error bars represent ±SD from three independent experiments. Statistical significance was determined by comparison to the NC: **p* < 0.05, ***p* < 0.01.


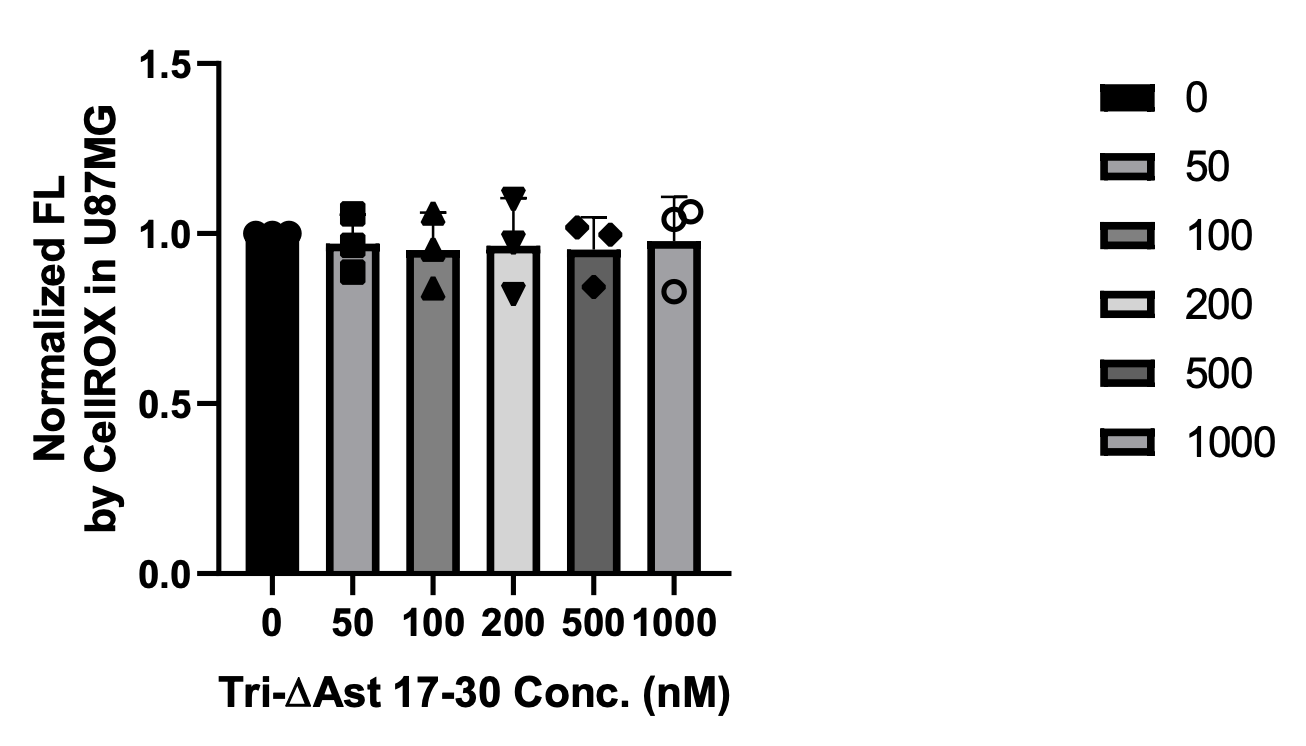


**Fig. S15**. Intracellular ROS quantification in U87MG cells following Tri-ΔAst17-30 aptamer treatment. CellROX assays were conducted after exposing cells to aptamer concentrations ranging from 0 to 1 µM for 24 h. No significant increase in intracellular ROS levels was detected under all tested concentrations, indicating that Tri-ΔAst17-30 does not induce oxidative stress in U87MG cells within the examined concentration range.


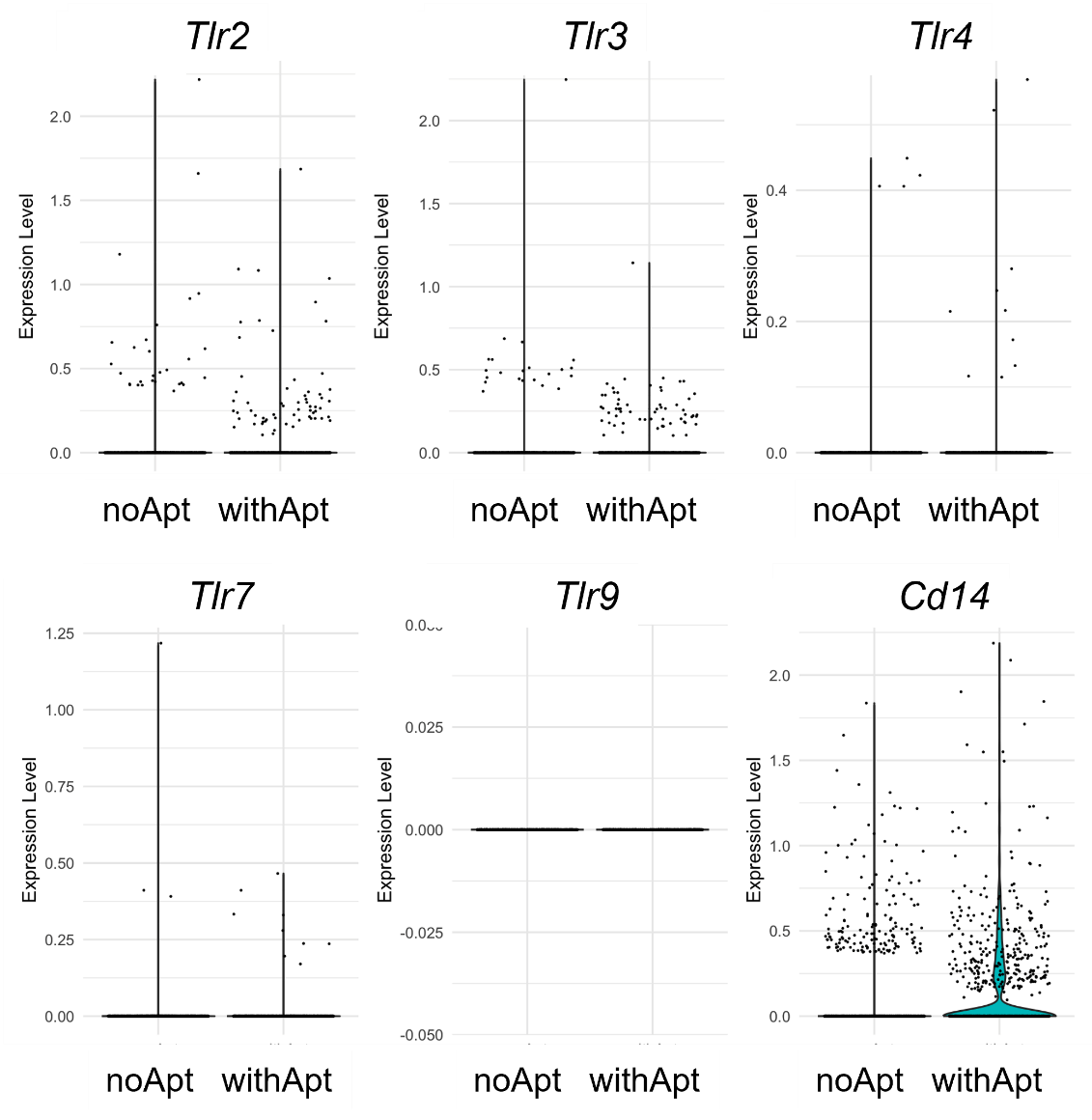


**Fig. S16**. Comparison of surface protein gene expression profiles potentially associated with ssDNA aptamer-triggered TNF–NF-κB activation in astrocyte-enriched clusters without (noApt) or with the Tri-ΔAst17-30 aptamer (withApt). The Wilcoxon rank-sum test was used to compare the noApt and withApt groups. Among the analyzed six genes, *Cd14* displayed the most significant differential expression (*p*=4.63×10⁻^10^, Wilcoxon rank-sum test).

**Table S1.** The SELEX protocol in each round

| **Round in SELEX** | **ssDNA Library conc.** [pmol] | **Differentiated Neurons^a)^** | | **Primary Astrocytes^b)^** | | **Washing times** | **ssDNA monitoring method** | **Use of MACS** |
| --- | --- | --- | --- | --- | --- | --- | --- | --- |
|  |  | **Cells** [mL^−1^] | **Library binding** | **Cells** [mL^−1^] | **Library binding** |  |  |  |
| **1** | 4×10^3^ | − | − | 5×10^6^ | 1 h | three | − | No |
| **2** | 345 | 5×10^6^ | 30 min | 5×10^6^ | 1 h | three | − | No |
| **3** | 80 | 5×10^6^ | 30 min | 5×10^6^ | 1 h | three | Absorbance | No |
| **4** | 80 | 5×10^6^ | 30 min | 5×10^6^ | 1 h | three | qPCR | No |
| **5** | 50 | 5×10^6^ | 45 min | 5×10^6^ | 45 min | three | Absorbance | No |
| **6** | 35 | 5×10^6^ | 45 min | 5×10^6^ | 45 min | three | Absorbance | No |
| **7** | 35 | 5×10^6^ | 45 min | 5×10^6^ | 45 min | three | qPCR | No |
| **8** | 35 | 5×10^6^ | 1 h | 5×10^6^ | 30 min | three | Absorbance | No |
| **9** | 35 | 5×10^6^ | 1 h | 5×10^6^ | 30 min | three | Absorbance | No |
| **10** | 35 | 5×10^6^ | 1 h | 5×10^6^ | 30 min | three | qPCR | No |
| **11** | 35 | 2.5×10^6^ | 30 min | 2.5×10^6^ | 30 min | three | Absorbance | No |
| **12** | 35 | 2.5×10^6^ | 30 min | 2.5×10^6^ | 30 min | three | Absorbance | No |
| **13** | 35 | 2.5×10^6^ | 30 min | 2.5×10^6^ | 30 min | three | qPCR | No |
| **14** | 35 | 5×10^6^ | 30 min | 5×10^6^ | 30 min | three | Absorbance | Yes |
| **15** | 35 | 2.5×10^6^ | 30 min | 2.5×10^6^ | 10 min | three | qPCR | Yes |
| **16** | 35 | 2.5×10^6^ | 30 min | 2.5×10^6^ | 10 min | three | qPCR | Yes |
| **17** | 35 | 2.5×10^6^ | 30 min | 2.5×10^6^ | 10 min | three | qPCR | Yes |
| **18** | 35 | 2.5×10^6^ | 30 min | 2.5×10^6^ | 10 min | three | qPCR | Yes |
| **19** | 35 | 2.5×10^6^ | 30 min | 2.5×10^6^ | 10 min | three | qPCR | Yes |
| **20** | 35 | 2.5×10^6^ | 60 min | 2.5×10^6^ | 10 min | three | qPCR | Yes |

^a)^Neurons were differentiated from primary rat cortical NPCs; ^b)^Astrocytes were isolated from primary rat brain cortex.

**Table S2.** Identification of ssDNA aptamer candidates during cell-SELEX

| **Group** | **ID*** | **Frequency****  **(%)** | **Sequence in the randomized region (5' to 3')** | **Length (nt)** |
| --- | --- | --- | --- | --- |
| 1 | 17-30  12-18 | 31 | TGC CGA AGG TGC CGA TTG AGT GAA TCG GCT GTT GTT TAT G  TGC CGA AGG TGC CGA TTG AGT GAA TCG GTT GTT GTT TAT G | 40  40 |
| 2 | 12-22  13-12  13-13  13-17  13-22  13-32  13-69  13-48 | 11 | GGC CGA CTT TCT TTT TTT CAT GTC TTA CGG GTT CTT TGT G  GGC CGG CTT TTT CTT TTC TTT ATA TTT TAT GGG TTC TCT GTG  GGC CGA CTT TCT CTT CTT TAT ATT TTA CGG GTT CTT TGT G  GGC CGA CTT TTT TTT TTT TTA TAT TTT ACG GGT CCT CTG TG  GGC CGA CTT TTT TTT TTT TAT ATT TTA CGG GTC CTC TGT G  GGC CAA TCT TTT TTT TTT ATA TTT TAC GGG TCC TTT GTG  GGC CAA CTT TTT TCT TTT TTA TAT TTA CGG GTC TTC TGT G  GGC CGA CTT TTT CTT TCT TAT ATT TTA CGT GTT TTT TGT G | 40  42  40  41  40  39  40  40 |
| 3 | 12-1  12-2  13-15  13-26  13-34  13-42  13-50  13-56 | 8 | CCA CAC TGA CAT TAT ATT TTA GCG GTT TTT TTT TTA TTC GTG  CCA CAC CGA CAT CAT ATT TCA GCA GTT TTT TTT ATT CGT G  CCA CAC CGA CTT ATA TTT CAG CGG TTT TTT TTT TTT GTT TGT G  CCA CAC CGA CTC ACA TTT CAG CAT TTT TTT TTA TTC GTG  CCA CAC TGA CAT CAT ATT TTA GCG GTT TTT TTT ATT CGT G  CCA CAC TGA CAT TAT ATT TTA GCG GTT TTT TTT ATT CGT G  CCA CAC CAA CAT CAT ATT TTA GCG GTT TTT TTT ATT TGT G  CCA CAC CAA CAT TAT ATT TTA GCG GTT TCT CTT ATT CGT G | 42  40  43  39  40  40  40  40 |
| 4 | 12-5  13-9  13-16  13-28  13-33  13-49  13-60 | 7 | CCA CTC GCC TTA TTA CAC CAT ATT GTT GCT TTT TTT GTG  CCA CCC GCT TTA TTA CAC TGG ACC GTT GCT TTT TTT TGT G  CCA CTC GCC TTA CCA CAT TAG ACC GTT GCT TTT TTT TGT G  CCA CTC GCC TTA CCA CAC TAT ACC GTT GCT TTT TTT TGT G  CCC TCG CTT TAT CAC ACC AGA CCG TTG TTT TTT TTT GTG  CCA CTT GCT TTA CCA CAC TAG ACC GTT GCT TTT TTT GTG  CCA CTC GCC TTA TTA CAC CAT ATT GTT GCT TTT TTC TGT G | 39  40  40  40  39  39  40 |

*The ID number (x-y) denotes the SELEX round (x) and the colony number during sequencing (y). **Frequency indicates the relative abundance of each sequence group of 130 total colonies. The three aptamers selected in the present study are marked in blue.

**Table S3.** DNA sequence used for experiments.

| **Name** | **Sequence (5**' **to** **3**'**)*** | | **Length (nt)** | **Modification** |
| --- | --- | --- | --- | --- |
| Library | *ATG CGG ATC CCG CGC*−N_40_−*CGC GCG AAG CTT GCG* | | 70 | 5' ALEXA488 |
| Forward | *ATG CGG ATC CCG CGC* | | 15 | - |
| Reverse | *CGC AAG CTT CGC GCG* | | 15 | - |
| Ast17-30 | *ATG CGG ATC CCG CGC* TGC CGA AGG TGC CGA TTG AGT GAA TCG GCT GTT GTT TAT G*CG CGC GAA GCT TGC G* | | 70 | 5' ALEXA488 |
| Ast17-30 scrambled | *ATG CGG ATC CCG CGC* GTA GTT GTG GTG GCT ACG CAT CGT ATC TGG TGA TCA GTG A*CG CGC GAA GCT TGC G* | | 70 | 5' ALEXA488 |
| ΔAst17-30 | CCG AAG GTG CCG ATT GAG TGA ATC GGC | | 27 | 5' ALEXA488 |
| ΔAst17-30 scrambled | GAA GCG CCG AGT ACT GCA GGC TAG GTT | | 27 | 5' ALEXA488 |
| Tri-ΔAst17-30 | Ya | GAC CGA TGG ATG ACC TGT CTG CCT AAT GTG CGT CGT AAG | 39 | - |
|  | Yb | GAC CGA TGG ATG ACT TAC GAC GCA CAA GGA GAT CAT GAG | 39 | - |
|  | Yc | GAC CGA TGG ATG ACT CAT GAT CTC CTT TAG GCA GAC AGG | 39 | - |
|  | Linker+ΔAst17-30 | T CAT CCA TCG GTC CCG AAG GTG CCG ATT GAG TGA ATC GGC | 13+27  =40 | 3' ALEXA488 |

*Forward and reverse primer regions are *italicized*.

**Table S4.** List of marker genes for each cell type

| **Cell Type** | **Cluster in UMAP** | | **Marker Genes** | **References** |
| --- | --- | --- | --- | --- |
| Astrocyte^a^ | | Cluster 0, 2, 9 | *Gfap, Aqp4, S100b, Ndrg2, Sox9, Aldoc, Slc1a3, Acsl6, Gsta4 , Gja1* | [29-35] |
| Astrocyte (Sox9−) | | Cluster 8 | *Gfap, Aqp4, S100b, Ndrg2, Aldoc, Slc1a3, Gsta4* | [29, 31, 32, 34] |
| Fetal Astrocyte | | Cluster 6 | *Top2a, Mki67, Rrm2, Ccna2* | [31, 36] |
| OPC | | Cluster 10 | *Pdgfra, Olig1, Olig2, Sox10, Epn2* | [37, 38] |
| Immature Neuron | | Cluster 5 | *Ascl1, Slc1a1, Olig1, Scg2, Nefl, Nefm, Reln* | [39-41] |
| Oligodendrocyte | | Cluster 4 | *Tubb4a, Sirt2, Cnp, Slain1, Plp1, Olig2, Mbp* | [37] |
| Neuron | | Cluster 1 | *Sox11, Gal, Igfbpl1, Nefl, Nefm* | [35] |
| Fibroblast | | Cluster 7 | *S100a6, Col3a1, Col6a1, Col1a1, Dcn* | [42] |
| Microglia | | Cluster 3 | *C1qa , Trem2, Aif1* | [43] |

^a^Astrocytes are clustered into three groups based on annotated gene expression levels: Cluster 0 (all genes expressed), Cluster 2 (low *Aqp4* and *Aldoc* expression), and Cluster 9 (moderate expression of all genes, but low *Aqp4* and *Gja1* expression).
